# Barriers to and Facilitators of Cervical Cancer Screening among Women in Southeast Asia: A Systematic Review

**DOI:** 10.3390/ijerph18094586

**Published:** 2021-04-26

**Authors:** Brandon Chua, Viva Ma, Caitlin Asjes, Ashley Lim, Mahsa Mohseni, Hwee Lin Wee

**Affiliations:** 1 Saw Swee Hock School of Public Health, National University of Singapore, 12 Science Drive 2, Singapore 117549, Singapore; e0639551@u.nus.edu (B.C.); mohseni_m63@yahoo.com (M.M.); 2 Health Economics and Outcomes Research Centre of Excellence (Greater Asia), Becton, Dickinson and Company, 2 International Business Park Road, Singapore 609930, Singapore; viva.ma@bd.com; 3 Government and Public Affairs, Becton, Dickinson and Company, 2 International Business Park Road, Singapore 609930, Singapore; caitlin.asjes@bd.com; 4 Department of Pharmacy, KK Women’s and Children’s Hospital, 100 Bukit Timah Road, Singapore 229899, Singapore; ashley.lim.sy@kkh.com.sg; 5 Faculty of Science, Department of Pharmacy, National University of Singapore, 18 Science Drive 4, Singapore 117543, Singapore

**Keywords:** cervical cancer screening, barriers, facilitators, southeast asia, pap smear, HPV test, visual inspection with acetic acid

## Abstract

In Southeast Asia, cervical cancer is the second most common cancer in women. Low coverage for cervical cancer screening (CCS) becomes a roadblock to disease detection and treatment. Existing reviews on CCS have limited insights into the barriers and facilitators for SEA. Hence, this study aims to identify key barriers and facilitators among women living in SEA. A systematic literature review was conducted on Pubmed, Embase, PsycINFO, CINAHL, and SCOPUS. Primary qualitative and quantitative studies published in English that reported barriers and facilitators to CCS were included. The Mix Methods Appraisal Tool was used for the quality assessment of the included studies. Among the 93 included studies, pap smears (73.1%) were the most common screening modality. A majority of the studies were from Malaysia (35.5%). No studies were from Timor-Leste and the Philippines. The most common barriers were embarrassment (number of articles, *n* = 33), time constraints (*n* = 27), and poor knowledge of screening (*n* = 27). The most common facilitators were related to age (*n* = 21), receiving advice from healthcare workers (*n* = 17), and education status (*n* = 11). Findings from this review may inform health policy makers in developing effective cervical cancer screening programs in SEA countries.

## 1. Introduction

In 2018, approximately 570,000 women developed cervical cancer and 311,000 women died from it [1]. Approximately 84% of all cervical cancers and 88% of all deaths caused by cervical cancer occurred in lower-resource countries [1]. Over the past four decades, a significant reduction in mortality and incidence of cervical cancer have been observed with preventive strategies such as cervical cancer screening (CCS) and vaccination against the human papilloma virus (HPV) [2]. Screening modalities for cervical cancer include a pap smear, HPV test, and visual inspection with acetic acid (VIA). Despite the proven effectiveness of screening, worldwide coverage of these preventive strategies remains poor, especially in developing countries [3].

The Southeast Asia (SEA) region comprises of 11 countries of diverse religions, cultures, and history: Brunei, Cambodia, Indonesia, Laos, Malaysia, Myanmar, Philippines, Singapore, Timor-Leste, and Vietnam. There are approximately 330 million women in SEA, equivalent to 4.3% of the world’s population [4]. Cervical cancer is the second most common cancer among women in the region [5]. In 2020, SEA was ranked seventh for cervical cancer incidence and sixth for mortality compared to other regions of the world [6]. Given the significant disease burden of cervical cancer in the presence of effective preventive strategies, a more detailed understanding of the barriers and facilitators to screening is needed to help in the planning of interventions to improve participation in screening.

Factors that influence CCS uptake include education status [7], health literacy [8], psychosocial factors [9], and contextual factors [10]. However, no study from SEA was included in these existing reviews. In addition, facilitators to CCS in countries with high disease incidence, including SEA nations, are not well characterized based on a systematic literature review [11]. Therefore, we aim to identify barriers to and facilitators of cervical cancer screening among women living in SEA, as well as to generate country-specific insights.

## 2. Materials and Methods

### 2.1. Search Strategy and Selection Criteria

The systematic review process was based on the Preferred Reporting Items for Systematic Reviews and Meta-Analyses (PRISMA) statement [12]. We searched in PubMed, Embase, CINAHL, SCOPUS, and PsycINFO to identify studies for inclusion without any date restrictions. The search strategies involved keywords and controlled vocabulary related to the concepts of CCS, SEA, barriers, and facilitators. The search was conducted on 3 November 2020 and full details of the search strategy can be found in Table S1.

Studies were included in this review if they met the following criteria: (1) Primary quantitative and qualitative research that reported barriers or facilitators of CCS uptake or intention, (2) involved participants living in SEA, (3) reported barriers or facilitators of CCS separately if more than one disease was analyzed, and (4) published in the English language. The exclusion criteria were studies that: (1) Compared diagnostic performance between different CCS modalities, (2) focused primarily on colposcopy, (3) reported screening as part of a screen-and-treat program, and (4) investigated an intervention to increase CCS uptake or intention without reporting any baseline barrier to or facilitator of CCS. Where the same cohort of patients were analyzed by different studies, only the latest publication was included for this review.

### 2.2. Data Collection and Analysis

Two independent reviewers (BC and AL) performed the search strategy and study selection, while two independent reviewers (BC and MM) executed the data extraction and quality assessment of the included studies. A manual search of the reference list for all included studies was done to identify additional studies for inclusion. All citations were uploaded on Endnote for the removal of duplicates and exported to Microsoft Excel for screening studies for inclusion.

The following information were abstracted from eligible articles: Study title, authors, publication year, study design, screening instrument used, population size, age of study participants, and proportion of patients with history of CCS. In addition, for quantitative studies, data extracted included barriers and facilitators that were statistically significantly associated (*p* < 0.05) with CCS intention or uptake, as well as proportions of participants reporting a barrier or facilitator. When univariate and multivariable analyses were both conducted, only results from multivariable analyses were extracted. For qualitative studies, data extracted included all reported barriers and facilitators.

Thematic analysis with an inductive approach was performed to classify barriers and facilitators into major categories [13], with a focus on context and commonalities across included studies. Data extracted from included studies were first assigned a code and patterns were searched amongst the coded data. Similar codes were subsequently categorized into descriptive themes, and themes were clustered into higher-ranking themes (major categories subsuming the themes). Finally, the number of studies supporting each theme were summed up for each SEA country. The Mixed Methods Appraisal tool (MMAT) was used to conduct a quality assessment of the included studies [14]. The MMAT has different scoring criteria for different types of studies: Mixed methods, qualitative, quantitative non-randomized, and quantitative descriptive. Each type of study was assessed based on five criteria with a “yes”, “no”, or “unsure” response, with a maximum score of 5. Any discrepancies for study inclusion, data extraction, analysis, and quality assessment were resolved through consensus or by referral to a third reviewer (VM or WHL).

## 3. Results

**Figure 1:**
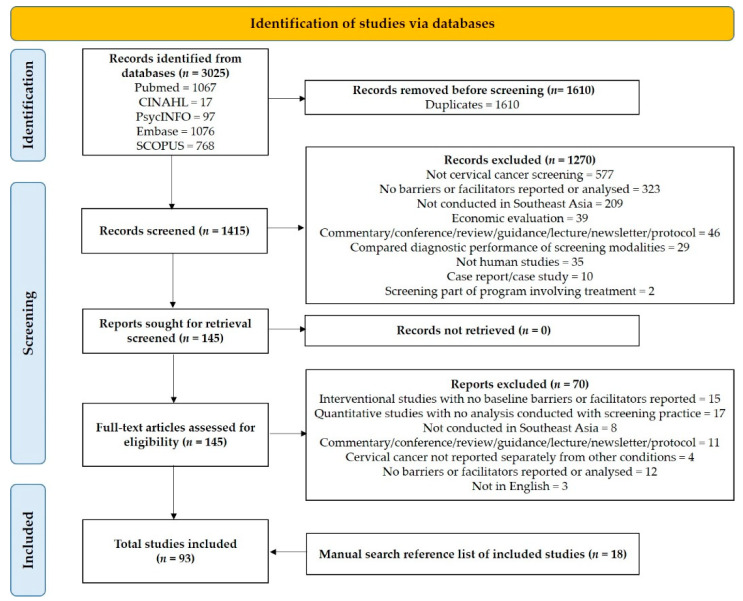
Preferred Reporting Items for Systematic Reviews and Meta-Analyses (PRISMA) flow diagram of included studies.

A total of 3025 records were retrieved from the databases. After the removal of 1610 duplicates, 1415 records underwent title and abstract screening resulting in the exclusion of 1270 articles (Figure 1). After a full text review of 145 studies, 75 articles met the inclusion criteria. In addition, we identified 18 studies that met inclusion criteria from manual search. Therefore, the final number of original research articles for data extraction, analysis, and synthesis was 93.

### 3.1. Study Characteristics

The study characteristics are detailed in Table 1. A total of 69 of the 93 studies were published within the past decade (2011 to 2020). A majority of the studies (*n* = 81) were cross-sectional or case-control studies, while the rest were qualitative (*n* = 9) or mixed-methods (*n* = 3) studies. Most of the studies were conducted in Malaysia (35.5%, *n* = 33) [15,16,17,18,19,20,21,22,23,24,25,26,27,28,29,30,31,32,33,34,35,36,37,38,39,40,41,42,43,44,45,46,47], followed by Thailand (24.7%, *n* = 23) [48,49,50,51,52,53,54,55,56,57,58,59,60,61,62,63,64,65,66,67,68,69,70], Indonesia (16.1%, *n* = 15) [71,72,73,74,75,76,77,78,79,80,81,82,83,84,85], and Singapore (15.1%, *n* = 14) [86,87,88,89,90,91,92,93,94,95,96,97,98,99]. None of the studies were conducted in the Philippines and Timor-Leste.

The number of study participants ranged from 7 to 15,074,126. Data were primarily obtained via the administration of questionnaires or interview or a combination of both. Pap smear was the screening modality in a majority of the studies (73.1%, *n* = 68), followed by VIA (11.8%, *n* = 11). A small number of studies involved self-sampled HPV tests alone (*n* = 3), and a combination of pap smears and VIA/self-sampled HPV tests (*n* = 6). Excluding studies that only recruited patients with no history of CCS, the prevalence of patients reporting a screening history varied widely from 3.9% in Laos [103] to 89.8% in Thailand [57]. In the two studies, none of the factors investigated were statistically significant [21,36], and thus were not included in the categorization of barriers and facilitators. As two studies in Thailand referred to the Reproduction Health Survey 2009 [62,70], data were only extracted from the more recent publication [70]. Two studies from Singapore analyzed the same study cohort but reported unique barriers and facilitators separately for each study [89,90]. Hence, both studies were included in this analysis.

### 3.2. Quality Assessment

A majority of the studies (68.8%, *n* = 64) scored 4 or 5 out of 5 based on the MMAT. All qualitative (*n* = 9) and mixed-methods studies (*n* = 3) scored either 4 or 5 points. Among 73 quantitative non-randomized studies, the lack of description of the sampling process or population (*n* = 38), and the lack of description of tools for an outcome and exposure measurement (*n* = 20) were common. Four studies were not clear on the completeness of screening data. Among eight quantitative descriptive studies, five studies lack description on sampling or target population, where non-response bias becomes difficult to assess. The complete quality appraisal can be found in Table S2.

### 3.3. Factors Associated with Cervical Cancer Screening in Southeast Asia (n = 91)

**Figure 2:**
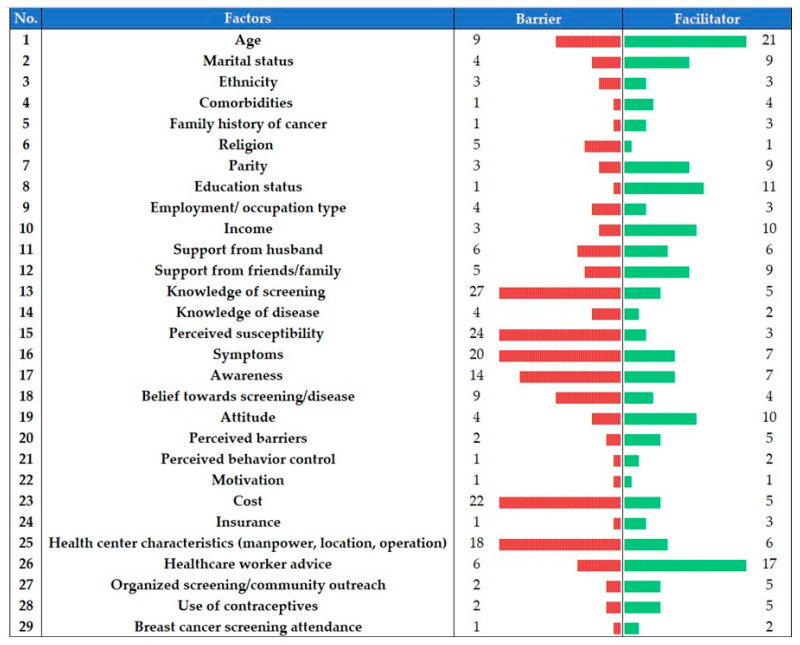
Factors described as barriers and facilitators of cervical cancer screening in Southeast Asia, according to publication numbers.

A total of 63 barriers from 63 studies were reported across seven countries in SEA (Table S3), while 71 facilitators from 73 studies were reported across nine countries (Table S4). The top three barriers to CCS in SEA by the number of publications include embarrassment (*n* = 33), busyness or time constraints (*n* = 27), and poor knowledge of screening (*n* = 27). The top three CCS facilitators include age (*n* = 21), healthcare workers’ advice for CCS (*n* = 17), and higher education status (*n* = 11). Figure 2 summarizes 29 factors that were described both as barriers and facilitators of CCS across studies in SEA (full details by country level are available in Figure S1).

The barriers to and facilitators of CCS were broadly organized into 11 categories: (1) Demographics, (2) socio-economic status, (3) healthcare utilization, (4) social support, (5) psychological, emotional, (6) knowledge, (7) risk perception, (8) perception, attitude, belief, (9) motivation, preference, (10) financial access, and (11) health system. Factors that do not fall under the 11 categorizes were classified as “others”.

### 3.4. Common Barriers and Facilitators in Cervical Cancer Screening Across Countries in Southeast Asia

The barriers to and facilitators of CCS were also summarized by the number of countries in Tables S3 and S4, respectively, to allow for commonalities across countries to be drawn. The barrier categories reported by most countries (*n* = 6) were demographics, knowledge, risk perception, and health system. Poor awareness to screening was the most common barrier reported by countries in SEA (*n* = 6). Other common barriers (*n* = 5) include poor knowledge of screening, poor perceived susceptibility, having no symptoms, factors related to health center characteristics (manpower, operations, and location), embarrassment, fear of results, fear of pain, and costs related to CCS. Most countries in SEA (*n* = 7) reported facilitators in the category of demographics. This is followed by knowledge, financial access, and health system-related facilitators (*n* = 6). Specific facilitators common across countries include age (*n* = 6), followed by good awareness of screening, and the receipt of healthcare worker advice (*n* = 5).

The commonalities among the top three barriers and facilitators for each country were also assessed. Embarrassment (*n* = 4) and poor knowledge to screening (*n* = 3) were the two most common barriers in SEA. Other common barriers (*n* = 2) include time constraints, having no symptoms, and the cost of screening. Age and advice from healthcare workers were the most common facilitators of CCS (*n* = 3), followed by health center characteristics (manpower, operations, and location), and support from friends or family members (*n* = 2).

### 3.5. Factors Associated with Cervical Cancer Screening (Country Level Analyses)

#### 3.5.1. Brunei (*n* = 1) 

Based on a population health survey among adults aged 18 to 69 years old, 56.5% of female participants had a history of having a pap smear [100]. Barriers to CCS include older age, employment type, and breast cancer screening attendance [100]. Facilitators to CCS include being married, the presence of comorbidities or family members with comorbidities, diet, and alcohol intake [100].

#### 3.5.2. Cambodia (*n* = 1)

Among women aged 20 to 69 in a rural district, only 7.0% have ever received a pap smear [101]. Only facilitators were reported in this cross-sectional study, where younger women, and those with good awareness towards screening expressed greater willingness to CCS [101].

#### 3.5.3. Indonesia (*n* = 15)

Two qualitative studies and thirteen quantitative studies were published after 2010, 11 of which reported VIA as the screening modality for CCS. Two studies reported pap smears for CCS [72,73], while two studies reported both pap smear and VIA [81,82]. The prevalence of having a screening done in the past ranged from 5.5% to 33.3% [71,72,73,76,80,81,85], while participants’ desire to screen was 45.2% and 57.4% based on two studies [77,84].

Of the seven studies reporting barriers to CCS in Indonesia, four studies reported facilitators in the categories of knowledge as well as perception, attitude, and belief. No studies reported barriers related to demographics or socio-economic status. Embarrassment [74,81], knowledge deficits in CCS [81,84], having no symptoms [74,81], and fear of result [76,81], were among the top barriers in Indonesia. Two studies described the lack of knowledge of cervical cancer as a barrier to CCS, which was only reported in Indonesia [74,81].

Facilitators were more commonly described in the categories of perception, attitude, and beliefs (*n* = 7) as well as motivation and perception (*n* = 5) among 13 studies from Indonesia. There were no facilitators in the classification of psychological or emotional factors, and healthcare utilization. Intentions to screening was among the top facilitators of CCS [72,78,80], which was reported more commonly in Indonesia compared to other SEA countries. In addition, support from family or friends was another crucial facilitator of CCS based on three publications [71,74,81].

#### 3.5.4. Laos (*n* = 3)

All three quantitative studies from Laos described pap smears as the screening modality of interest, two of which were published after 2010 [103,104]. Among working women, a higher prevalence of ever receiving a pap smear was reported (46.3%) [104], compared to village residents and women with HIV (3.9% to 4.5%) [102,103].

All three studies reported barriers in the category of psychological or emotional factors, knowledge, risk perception, financial access, and the health system. No studies reported barriers in the category of socio-economic status. Barriers reported by at least two studies include embarrassment [102,103,104], fear of pain [102,103,104], fear of results [102,103], poor awareness [103,104], poor perceived susceptibility [102,103], being not at risk for cancer [103,104], and having no symptoms [103,104]. Other barriers include concerns with screening cost [102,103,104], an inaccessible screening location [102,103], and the lack of CCS advice from a healthcare worker [103,104]. Only one study described a knowledge-related facilitator, where higher scores for knowledge of cervical cancer and prevention were reported among women who received CCS in the past [104].

#### 3.5.5. Malaysia (*n* = 31)

Nineteen studies were published from 2011, five of which were qualitative studies. The take up of pap smear in different female populations varied largely, from 6.0% among university students [18], to 79.5% among patients from an obstetrics and gynecology clinic [47]. Similarly, screening in the past three years varied largely from 3.8% among African immigrants to Malaysia [22], to 42.8% among university staff [38].

Across 21 studies in Malaysia reporting barriers to CCS, a majority of the studies reported barriers in the categories of psychological or emotional factors (*n* = 16) and knowledge (*n* = 13). Embarrassment was the most common reason cited for not attending CCS [15,17,18,20,23,29,30,33,38,39,46,47] followed by having no time or busyness [16,17,20,23,25,30,31,33,38,39,46]. Other top barriers include fear of pain [17,18,19,20,29,31,33,47], and poor knowledge of screening [16,18,20,31,33,38,39,46,47]. Barriers relating to health center characteristics were reported in eight studies [17,20,30,31,33,38,39,46]. This includes a long wait time for screening [30,31,38], an inconvenient screening location [17,33,46], difficulty in securing an appointment for screening [31], inconvenient clinic hours [30,31], having no adequate facility for screening [46], no transport [39], and no female health provider [17,20,30,33]. Compared to other nations in SEA, worry [18,20,33] and the lack of support from their husband [18,20,23], friends, or family [30,33,38,46], were prominent barriers to CCS in Malaysia.

A total of 26 studies reported facilitators to CCS in Malaysia, a majority of which involved facilitators categorized under demographics (n = 15) and knowledge (n = 11). Among the top facilitators were demographic factors such as age [24,25,32,34,37,39,40,43,44,46], marriage status [22,24,33,37,40,43], and parity [15,32,38,43,44]. Receiving advice from healthcare workers [19,27,29,30,33,47], and a good attitude towards CCS [26,35,37,38,41], were also common facilitators reported in Malaysia. Other facilitators, which were also more commonly reported in Malaysia than other SEA nations, include good knowledge of screening or cervical cancer [22,24,26,32,35,37,41,43,44], and the use of contraceptives [24,27,37,41,44].

#### 3.5.6. Myanmar (*n* = 1)

A total of 666 migrant women were surveyed in a study, where 19.1% had CCS in the past three years [105]. Screening in the past three years was more likely among older women, those with a family history of cancer, had expressed willingness to pay for screening, received encouragement from nurses, had low perceived barriers, and had good disease and screening knowledge [105].

#### 3.5.7. Singapore (*n* = 14)

Of the 11 quantitative, one qualitative and two mixed-methods studies, nine were published between 2011 to 2020. The prevalence of ever being screened for cervical cancer varied widely from 2.8% among undergraduates [91], to 69.2% among participants in the National Health Survey [98]. Similarly, the proportion of patients who received CCS within the past three years varied widely from 2.9% to 44.3% [86,87,88,89,92,93,94,95,97,98].

Seven of the ten studies reporting barriers to CCS in Singapore described barriers classified under risk perception and motivation or preference. Six studies reported barriers in the categories of financial access and psychological or emotional factors. Perceived susceptibility was the most common barrier to CCS, where patients do not care about screening [87,92,93,94,95,96,98]. Other top barriers to CCS include busyness or lack of time [91,92,93,94,95,96], cost concerns relating to screening [87,93,94,95,96,98], embarrassment [92,93,94,95], and the fear of receiving unfavorable results [87,93,95,96]. Amongst the top barriers was the poor knowledge of screening which involved knowledge of a screening location [94,95], screening frequency [95], and screening criteria [87,94,95,96]. Barriers more commonly reported in Singapore compared to other SEA nations, include having a fatalistic attitude [88,94,95,96], inconvenience [94,95,96], and the lack of companions to attend screening with [94,95,96].

Of the 12 studies reporting facilitators in Singapore, five reported facilitators within the category of perception, attitude, and belief, while four described facilitators in the categories of knowledge, demographics, and healthcare utilization. Top facilitating factors were related to age [89,92,99], support from family and friends [88,89,92], good awareness of screening [89,92,96], and high perceived benefit [90,92,99]. Other top facilitators include healthcare worker advice for screening [89,92,96] and health center characteristics such as a convenient screening location [87,93,96] and the availability of female health workers or nurses for the conduct of screening [87,96].

#### 3.5.8. Thailand (*n* = 23)

A majority of the studies (*n* = 17) were published between 2011 to 2020. The prevalence of ever having pap smear screening ranged between 36.6% to 89.8% [48,49,50,51,53,57,59,64,66,69,70], while screening with pap smear or VIA in the past five years ranged from 32.5% to 85.8% [52,54,58,61,62]. Most of the studies described pap smears as the screening modality while one study explored the willingness to screen with a self-HPV test [67].

A majority of the 19 studies reporting barriers in Thailand described barriers classified under psychological or emotional factors (*n* = 15) and risk perception (*n* = 13). Similar to Malaysia, embarrassment was the most common reason for not receiving CCS in Thailand [48,49,50,51,53,57,58,59,60,62,69,70]. This is followed by poor knowledge of screening [48,50,51,53,61,62,63,67,68,70], having no symptoms [48,49,50,53,54,58,59,61,62,68,69], busyness or the lack of time [50,53,54,57,58,59,62,63,70], fear of pain [48,49,50,53,58,63,69,70], and poor perceived susceptibility [48,50,51,54,58,62,69,70]. Besides that, religion [48,53,62,70], and having a poor impression of the health system [56,58,60,63,69], were reported more frequently as barriers to CCS in Thailand.

Of the 17 studies reporting facilitators, nine described facilitators in the health system category, while eight reported demographic-related facilitators. The top facilitator of CCS was receiving advice from healthcare workers [48,53,54,56,61,69]. This is followed by demographic and socio-economic factors such as age [50,64,65,69,70], income level [54,56,68,70], and education status [53,62,64,70]. Having gynecological symptoms were amongst the top facilitators and was also more commonly reported in Thailand compared to other countries [50,53,54,66,68]. Other facilitators more commonly reported in Thailand include CCS being part of a routine health check [50,53,54,56,66], advice from employers [50,54,61], occupation type [52,70], and the fear or suspicion of cervical cancer [50,53,61].

#### 3.5.9. Vietnam (*n* = 2)

A qualitative and a mixed methods study with a total of 216 participants revealed a low prevalence of prior pap smear or VIA ranging from 3.1% to 7.1% [106,107]. Barriers to CCS were related to religious beliefs, low risk perception, and the lack of healthcare worker advice [106]. Both studies reported poor awareness as a barrier to screening [106,107], while the beliefs of health workers that CCS is only for married women was reported in one study [107]. Facilitators to CCS include spousal support, low cost of screening, and having healthcare workers’ advice for CCS [106].

## 4. Discussion

To our knowledge, this is the first systematic review to focus on barriers to and facilitators of CCS among women in SEA, a region with high disease incidence yet poor screening uptake. Over 60 barriers from seven countries and over 70 barriers from nine countries were identified and categorized into 11 broad categories. We presented the findings at the country level to provide insights on how screening uptake can be improved in each country. We also compared the top barriers and facilitators between countries, to provide guidance for countries that have limited information on factors affecting CCS. A majority of the studies in this review were published within the past 10 years, which highlights the growing interest and challenges faced in the area of cervical cancer screening within SEA. A broad search strategy was employed in this study to maximize findings by including both quantitative and qualitative studies without any date restrictions. This allowed for the study of a broader perspective from patients, family members, healthcare providers, and health officials, which provides valuable insight to guide the design of public health programs.

The barriers and facilitators identified from this study are related to demographics, socio-economic status, social support, knowledge, attitudes, perceptions, financial access, health system, and psychological or emotional factors. We found that the top barrier category to CCS is psychological or emotional factors (*n* = 44), namely embarrassment and fear. This is followed by knowledge (*n* = 38), which includes the lack of knowledge and awareness to cervical cancer and CCS. The top facilitator categories are predominantly factors related to demographics (*n* = 33), as well as perception, attitudes, and beliefs to screening (*n* = 29). These are consistent with prior research in lower-middle income countries [108,109,110], and interestingly in developed countries as well, such as the United States [111] and Australia [112]. Our findings also support a previously demonstrated relationship between higher education status and higher CCS uptake [7]. Similarly, psychosocial and contextual factors described in prior systematic literature reviews [9,10] were also reported by women in SEA. These include factors associated with the health system, cost, time constraints, screening attitudes, knowledge, awareness, emotional factors, social support, and experiences with healthcare professionals. However, our findings differed from a review among high cervical cancer incidence countries where the top barriers to CCS were fatalism, and negative attitudes and beliefs towards non-traditional healthcare [11]. A possible reason is that a majority of the studies included in that review are from Africa, where traditional healers are likely sought prior to or in conjunction with medical care [113].

Barriers common to SEA countries, reported by five to six countries, include poor awareness and knowledge of screening, poor perceived susceptibility to cervical cancer, having no symptoms, and factors related to health center characteristics. Facilitators common across countries include the influence of age, receiving advice from healthcare workers, and good awareness of screening. These common factors identified can also provide guidance for countries with limited insights into barriers and facilitators to CCS, such as the Philippines, Timor-Leste, Cambodia, Myanmar, Vietnam, and Laos. Several factors were also unique in certain countries, which reported certain barriers and facilitators more frequently than other countries. For example, religion and poor impression of the health system were more frequently reported barriers in Thailand while occupation type, advice from employers, and receiving CCS as part of a structured health program were more frequently reported facilitators. In Malaysia, unique barriers include the lack of support from husband, family members, and friends, while unique facilitators include the knowledge of screening and the use of contraceptives. Reasons for these differences between countries could be driven by the social, cultural, religious, and health system differences of SEA countries [114], as well as researchers’ interests in specific factors affecting CCS in the country. Hence, existing CCS programs should consider addressing the country-specific barriers and facilitators in the design of interventions to increase screening uptake.

Broadly, our findings suggest that patient education-based interventions are key to increasing CCS uptake in SEA, as key barriers to CCS such as fear, embarrassment, and the lack of knowledge can be addressed. Through the use of community health workers, brochures, phone counselling, and multimedia, educational interventions alone has been found to increase the odds of CCS uptake by more than 2 times [115], compared to routine care. Equally important, facilitators of CCS should be considered in the design of such interventions to increase CCS uptake, as barriers and facilitators are often two sides of the same coin. For example, knowledge of screening is one of the 29 factors that was described both as a barrier and facilitator across studies in SEA. Improving knowledge to screening among women facilitates CCS uptake while the lack of it represents a barrier. While these factors may represent key targets for intervention design, their differential impact across various contexts have been carefully considered [116]. Furthermore, the experience of different facilitators may have a greater influence on CCS uptake in a setting where barriers are commonly experienced by women. In a UK study, women with up-to-date CCS prioritized the following facilitators of CCS compared to those who had overdue or never had CCS: (1) Perceived benefits of CCS and (2) perceived self-responsibility over one’s health [117]. On the other hand, the ranking of common barriers, such as fear and embarrassment did not differ by participants’ screening history. The interplay of barriers and facilitators to CCS warrants further research, and the findings can be harnessed to guide interventions to increase CCS uptake. Therefore, we have presented barriers to CCS alongside its facilitators at the country level in this study.

The current systemic literature review has a few limitations. Firstly, the availability of evidence varies in SEA countries. A majority of the studies identified in the current review are from upper-middle income countries according to the World Bank criteria (Indonesia, Malaysia, and Thailand) [118], and fewer are published from lower-middle income countries (Myanmar, Cambodia, Vietnam, and Laos). In addition, there was no study from the Philippines and Timor-Leste. Although the common themes identified across SEA countries can provide insights into barriers to and facilitators of CCS in lower-middle income countries in the region, we do think that country-specific studies are still necessary, owing to the cultural differences between countries. However, technical expertise may be lacking in some countries, which highlights the need for support from foundations or non-governmental organizations with interests in women’s health. Secondly, we have applied an English language restriction in this review, while relevant articles may have been published in local languages. However, we assess its impact to be low as few studies (*n* = 3) were not published in English. Thirdly, the top barriers and facilitators identified in this review was based on publication numbers, which are dependent on previous researchers’ study interest, as well as questionnaire or interview design, where selected factors may be explored more frequently. Nevertheless, this remains a common methodology employed to assess the importance of factors identified in barriers and facilitator reviews, especially when no acceptable standard practice guideline is available at present [116]. Lastly, the heterogeneity among studies limits us from assessing the effect size of each barrier or facilitator on CCS uptake. Such heterogenicity is anticipated and necessary, which highlights the need for culture-specific studies and interventions to address the diverse cultural contexts in SEA countries.

## 5. Conclusions

A significant number of barriers affect the uptake of cervical cancer screening. Coupled with a lack of resources and possibly low prioritization of cervical cancer, challenges to address these barriers remain. This is perhaps why women in SEA continue to face a high risk of cervical cancer mortality and morbidity while significant progress has been made in the developed world. This review of studies published in the SEA region from 1994 to 2020 identified that psychological-, emotional-, and knowledge-related factors remain a significant concern among women in SEA. Facilitating factors include receiving advice from healthcare workers for CCS, and factors associated to patient demographics. Future studies can explore the interplay of the barriers and facilitators on CCS and generate more insights into low-middle income countries in the region. Nevertheless, findings from this review may inform health policy makers in developing effective cervical cancer screening programs in SEA countries.

## Figures and Tables

**Table 1:** Characteristics of the included studies.

Author (Year)	Country	Study Design/Instrument Used	Sample Size	Age of Participants (Years)	Type of Screening Methods Used	Prevalence of Ever Receiving Screening in the Past	MMAT Score
Suhaimi et al. (2020) [100]	Brunei	Cross-sectional/interview with structured questionnaire	3808 (2131 females)	Mean (SD): 41.9 (14.5)	Pap smear	56.5%	5
Touch et al. (2018) [101]	Cambodia	Cross-sectional/interview with structured questionnaire	440	Distribution: 20–29: 88 (20.0%) 30–39: 88 (20.0%) 40–49: 88 (20.0%) 50–59: 88 (20.0%) 60–69: 88 (20.0%)	Pap smear	7.0%	5
Kim et al. (2012) [74]	Indonesia	Qualitative/interview and focus group discussion	87	Range: 25–50	VIA	Unspecified	5
Anggraeni et al. (2016) [72]	Indonesia	Cross-sectional/survey questionnaire	96	Distribution: <20 years: 2 (2.1%) 20–35 years: 44 (45.8%) >35 years: 50 (52.1%)	Pap smear	33.3%	4
Nurhasanah et al. (2017) [76]	Indonesia	Cross-sectional/unspecified	176	Distribution: 20–29 years: 35 (19.9%) 30–39 years: 51 (29.0%) 40–49 years: 71 (40.3%) 50–59 years: 19 (10.8%)	VIA	33.0%	3
Sidabutar et al. (2017) [79]	Indonesia	Cross-sectional/survey questionnaire	80	Unspecified	VIA	Unspecified	2
Wakhidah et al. (2017) [83]	Indonesia	Case-control/survey questionnaire	150	Unspecified	VIA	NA	4
Anwar et al. (2018) [73]	Indonesia	Cross-sectional/interview with structured questionnaire	5397	Mean: 52.9	Pap smear	5.5%	5
Winarti et al. (2018) [85]	Indonesia	Case-control/survey questionnaire	410	Unspecified	VIA	NA	3
Sidabutar et al. (2018) [80]	Indonesia	Cross-sectional/interview with structured questionnaire	245	Unspecified	VIA	15.5%	4
Aprina et al. (2018) [71]	Indonesia	Cross-sectional/survey questionnaire	361	Unspecified	VIA	26.9%	3
Rahmawati et al. (2018) [77]	Indonesia	Cross-sectional/survey questionnaire	188	Range: 20–55	VIA	Desire to screen: 57.4%	4
Saptowati et al. (2018) [78]	Indonesia	Cross-sectional/survey questionnaire	200	Unspecified	VIA	Unspecified	3
Sutarti et al. (2018) [82]	Indonesia	Cross-sectional/survey questionnaire	369	Unspecified	Pap smear, VIA	Unspecified	3
Spagnoletti et al. (2019) [81]	Indonesia	Qualitative/focus group discussion and interview	Focus group: 17 Interview: 22	Range: Focus group female: 28–40 Focus group male: 35–45 Interview female: 22–57	Pap smear, VIA	31.8%	4
Widayanti et al. (2020) [84]	Indonesia	Cross-sectional/interview	126	Distribution: <20: 9 (7.1%) 20-30: 72 (57.1%) 30-40: 45 (35.7%)	VIA	Willingness to screen: 45.2%	2
Muhith et al. (2020) [75]	Indonesia	Cross-sectional/survey questionnaire	393	Range: 20–50	VIA	Unspecified	2
Phongsavan et al. (2010) [102]	Laos	Cross-sectional/interview with structured questionnaire	800	Mean (SD): 34.0 (9.4)	Pap smear	4.5%	5
Sichanh et al. (2014) [103]	Laos	Case-control/interview with structured questionnaire	640	Mean (SD): 36.2 (8.0)	Pap smear	3.9%	5
Hando et al. (2018) [104]	Laos	Cross-sectional/interview with structured questionnaire	356	Mean (SD): 38.2 (9.8)	Pap smear	46.3%	3
Chee et al. (2003) [42]	Malaysia	Cross-sectional/survey questionnaire	486	Mean (SD): 26.7 (6.2)	Pap smear	6.4% Within past 3 years: 4.3%	2
Chee et al. (2003) [24]	Malaysia	Cross-sectional/survey questionnaire	1720	Mean (SD): 30.1 (7.9)	Pap smear	25.3% Within past 3 years: 18.4%	4
Asmani et al. (2007) [39]	Malaysia	Cross-sectional/survey questionnaire	280	Distribution: <30: 49 (17.5%) 30–39: 79 (28.2%) 40–49:78 (27.9%) >50: 74 (26.4%)	Pap smear	51.4%	4
Moy et al. (2007) [40]	Malaysia	Cross-sectional/survey questionnaire	112	Mean (SD): 35.8 (9.1)	Pap smear	61.6%	4
Wong et al. (2008) [30]	Malaysia	Qualitative/interview	20	Mean (range): 32.2 (21–56)	Pap smear	0%	4
Othman et al. (2009)	Malaysia	Cross-sectional/survey questionnaire	221	Mean (range): 51 (41–61)	Pap smear	51.8%	2
Wong et al. (2009) [45]	Malaysia	Qualitative/semi-structured interview	20	Mean (range): 32.2 (21–56)	Pap smear	0%	5
Abdullah et al. (2010) [16]	Malaysia	Qualitative/semi-structured interview	11 (providers)	Range: 37–57	Pap smear	Unspecified	5
Al-Naggar et al. (2010) [18]	Malaysia	Cross-sectional/survey questionnaire	285	Mean (SD): 20.9 (1.9)	Pap smear	6.0%	3
Al-Naggar et al. (2010) [17]	Malaysia	Qualitative/focus group discussion	23 (17 females)	Range: 22–26	Pap smear	Unspecified	5
Dunn et al. (2010) [46]	Malaysia	Cross-sectional/survey questionnaire	1013	Mean (SD): 42.9 (10.1)	Pap smear	63.0%	4
Oon et al. (2010) [47]	Malaysia	Qualitative/interview with structured questionnaire	52 (44 females)	Range: 23–70	Pap smear	79.5%	5
Abdullah et al. (2011) [15]	Malaysia	Cross-sectional/survey questionnaire	403	Distribution: <35: 203 >35: 200	Pap smear	38.0%	5
Al-Naggar et al. (2012) [33]	Malaysia	Cross-sectional/survey questionnaire	142	Mean (SD): 31.6 (8.2)	Pap smear	46.5%	3
Aziz et al. (2013) [32]	Malaysia	Cross-sectional/interview with structured questionnaire	3693	Mean (SD): 36.7 years (7.7)	Pap smear	Within past 3 years: 52.2%	4
Baskaran et al. (2013) [20]	Malaysia	Cross-sectional/interview with structured questionnaire	369	Mean (SD): 37.5 years (10.0)	Pap smear	75.6%	3
Gan et al. (2013) [44]	Malaysia	Cross-sectional/interview with structured questionnaire	959	Mean (SD): 45.2 (12.2)	Pap smear	48.9% Within past 3 years: 18.4%	5
Wong et al. (2013) [43]	Malaysia	Cross-sectional/survey questionnaire	231	Median (IQR): Tested: 46 (37–53) Not tested: 30.5 (25–43)	Pap smear	55.8%	4
Azrai et al. (2015) [29]	Malaysia	Cross-sectional/interview with structured questionnaire	98	Mean (SD): 42.9 (12.9)	Self-HPV test	78.6% (Pap smear)	3
Abdullah et al. (2016) [27]	Malaysia	Cross-sectional/interview with structured questionnaire	515	Mean (SD): 58.8 (7.1)	Pap smear	39.2%	5
Danial et al. (2016) [21]	Malaysia	Cross-sectional/survey questionnaire	337	Distribution: 18-30: 148 (43.9%) 31-40: 118 (35.0%) ≥41: 71 (21.1%)	Pap smear	32.9%	4
Ma’som et al. (2016) [25]	Malaysia	Cross-sectional/survey questionnaire	839	Median (IQR): 38 (30–48)	Pap smear	63.1%	3
Indra et al. (2017) [37]	Malaysia	Cross-sectional/interview with structured questionnaire	147	Range: 18–63	Pap smear	77.6%	4
Razi et al. (2017) [38]	Malaysia	Cross-sectional/survey questionnaire	187	Distribution: 20–29: 18 (9.6%) 30–39: 106 (56.7%) 40–49: 57 (30.5%) 50: 6 (3.2%)	Pap smear	65.2% Within past 3 years: 42.8%	4
Abdullah et al. (2018) [34]	Malaysia	Cross-sectional/survey questionnaire	164	Mean (SD): 40.6 (8.4)	Self-HPV test	73.2%	4
Nwabichie et al. (2018) [22]	Malaysia	Cross-sectional/survey questionnaire	320	Distribution: 18–30: 100 (31.3%) 31–50: 218 (68.1%) 51–69: 2 (0.6%)	Pap smear	27.5% Within past 3 years: 3.8%	5
Rubini et al. (2018) [31]	Malaysia	Cross-sectional/interview with structured questionnaire	305 (males and females)	Range: >18	Pap smear	Unspecified	4
Sundraraj et al. (2018) [26]	Malaysia	Cross-sectional/survey questionnaire	246	Unspecified	Pap smear	28.5%	2
Yunus et al. (2018) [36]	Malaysia	Cross-sectional/survey questionnaire	316	Mean (SD): 41.2 (9.2)	Pap smear	Every 3 years: 9.5% Within past 3 years: 41.8%	4
Romli et al. (2019) [41]	Malaysia	Cross-sectional/survey questionnaire	210	Mean (SD): 43.0 (10.3)	Pap smear	55.2% Within past 5 years: 38.6%	5
Siraj et al. (2019) [28]	Malaysia	Cross-sectional/survey questionnaire	300	Distribution 17–40: 71 (23.7%) 41–50: 64 (21.3%) 51–60: 80 (26.7%) >60: 85 (28.3%)	Pap smear	57.0%	4
Baharum et al. (2020) [19]	Malaysia	Cross-sectional/survey questionnaire	417	Mean (SD): 24.9 (3.6)	Pap smear	Unspecified	5
Ting et al. (2020) [35]	Malaysia	Cross-sectional/self-administered online questionnaire	246	Distribution: 20–30: 141 (57.3%) 31–40: 65 (26.4%) 41–50: 18 (7.3%) 51–60: 16 (6.5%) 61–70: 6 (2.4%)	Pap smear	48.0%	4
Nandar et al. (2015) [105]	Myanmar	Cross-sectional/interview with structured questionnaire	666	Distribution: 30–39: 421 (64.1%) 40–49: 236 (35.9%)	Unspecified	Within past 3 years: 19.1%	5
Seow et al. (1994) [89]	Singapore	Cross-sectional/survey questionnaire	568	Distribution: 21–29: 65 (11.7%) 30–39: 189 (33.4%) 40–49: 152 (26.9%) 50–59: 126 (22.1%) 60–65: 34 (6.0%)	Pap smear	54.4%	5
Seow et al. (1995) [90]	Singapore	Cross-sectional/interview with structured questionnaire	568	Distribution: 21- 29: 65 (11.7%) 30–39: 189 (33.4%) 40–49: 152 (26.9%) 50–59: 126 (22.1%) 60–65: 34 (6.0%)	Pap smear	Unspecified	5
Seow et al. (2000) [88]	Singapore	Cross-sectional/interview with structured questionnaire	447	Range: 45–69	Pap smear	52.5% Within past 3 years: 34.9%	5
Lee et al. (2002) [87]	Singapore	Cross-sectional/interview with structured questionnaire	726	Range: 30–59	Pap smear	62.1% Every 3 years: 41.6%	5
Wee et al. (2010) [94]	Singapore	Cross-sectional/survey questionnaire	213 (125 females)	Median (IQR): 63.0 (49–76)	Pap smear	Eligible for screening every 3 years and went: 2.9%	4
Wee et al. (2012) [95]	Singapore	Cross-sectional/survey questionnaire	1081 (623 females)	Distribution: 40–50: 257 (23.8%) 50–60: 301 (27.8%) 60–70: 192 (17.8%) ≥70: 331 (30.6%)	Pap smear	Eligible for screening every 3 years and went: 31.3%	4
Shea et al. (2013) [91]	Singapore	Cross-sectional/survey questionnaire	393	Mean (SD): 21.1 (1.4)	Pap smear	2.8%	3
Chirayil et al. (2014) [86]	Singapore	Cross-sectional/survey questionnaire	206	Range: 18–26	Pap smear	5.3% Within past 3 years: 3.9%	3
Wong et al. (2015) [98]	Singapore	Cross-sectional/survey questionnaire	4337 (1993 eligible for cervical cancer screening)	Range: 18–79	Pap smear	69.2% Every 3 years: 43.8%	5
Tay et al. (2015) [92]	Singapore	Cross-sectional/survey questionnaire	1622	Distribution: Not stated: 22 (1.4%) <25: 445 (27.4%) 25–29: 456 (28.1%) 30–34: 220 (13.6%) 35–39: 121 (7.5%) 40–44: 112 (6.9%) 45–49: 86 (5.3%) 50–54: 86 (5.3%) >54: 74 (4.6%)	Unspecified	50.2% Within past 3 years: 21.2%	3
Wee et al. (2016) [93]	Singapore	Mixed method/survey questionnaire and interview	1996 (1154 females) /20 interviewed	Distribution: <60 years: 964 (48.3%) ≥60 years: 1032 (51.7%)	Pap smear	Every 3 years (rental flats): 18.0%	4
Wee et al. (2016) [96]	Singapore	Qualitative/interview	29 (20 patients, 9 provider)	Range of patient group: 40–59: 11 (55.0%) ≥60: 9 (45.0%)	Pap smear	Unspecified	5
Wee et al. (2017) [97]	Singapore	Mixed method/survey questionnaire and interview	2037 (855 females)/12 (6 females)	Distribution: 40–50: 412 (20.2%) 51–60: 1625 (79.8%)	Pap smear	Every 3 years: 24.9%	5
Yeo et al. (2018) [99]	Singapore	Cross-sectional/survey questionnaire	268	Distribution: 21–34: 208 (77.9%) 35–50: 59 (22.1%)	Pap smear	38.7%	4
Boonmongkon et al. (2002) [68]	Thailand	Cross-sectional/survey questionnaire	1028	Unspecified	Pap smear	Within past 2 years: 34.6%	2
Kritpetcharat et al. (2003) [51]	Thailand	Cross-sectional/interview with structured questionnaire	1199	Distribution: 20–29: 185 (15.4%) 30–39: 345 (28.8%) 40–49: 315 (26.3%) 50–59: 207 (17.3%) ≥60: 147 (12.3%)	Pap smear	66.9%	3
Boonpongmanee et al. (2007) [64]	Thailand	Cross-sectional/survey questionnaire	189	Mean (SD): 36.8 (7.69)	Pap smear	51.9%	3
Chalapati et al. (2007) [49]	Thailand	Prospective quasi-experimental/interview	200	Mean Intervention: 47.0 Control: 47.4	Pap smear	72.5%	5
Kietpeerakool et al. (2009) [61]	Thailand	Cross-sectional/interview with structured questionnaire	402	Mean (SD): 27.1 (6.6)	Pap smear	85.8%	3
Oranratanaphan et al. (2010) [59]	Thailand	Cross-sectional/survey questionnaire	78	Mean: 32.5	Pap smear	79.5%	2
Srisakul et al. (2011) [55]	Thailand	Cross-sectional/interview with structured questionnaire	450	Mean (SD): 45.3 (4.2)	Unspecified	Unspecified	3
Chesun et al. (2012) [56]	Thailand	Case-control/interview with structured questionnaire	400	Mean (SD): Case: 42.1 (5.3) Control: 42.5 (5.0)	Pap smear	Unspecified	4
Thanapprapasr et al. (2012) [50]	Thailand	Cross-sectional/survey questionnaire	1365	Distribution: <30: 713 (53.6%) 31–40: 384 (28.9%) 41–50: 159 (12.0%) ≥50: 73 (5.5%)	Pap smear	36.6%	4
Budkaew et al. (2014) [54]	Thailand	Case-control/survey questionnaire and interview	195	Mean (range): 46.0 (30–60)	Pap smear	NA	3
Oranratanaphan et al. (2014) [66]	Thailand	Cross-sectional/survey questionnaire	100	Mean (SD): 40.6 (9.3)	Self-HPV test, pap smear	81.0%	2
Wongwatcharanukul et al. (2014) [53]	Thailand	Cross-sectional/interview with structured questionnaire	547	Mean (SD): 43.0 (8.6)	Pap smear	64.9%	4
Mukem et al. (2015) [62]	Thailand	Cross-sectional/survey questionnaire	Health and Welfare Survey: 11,046,520	Mean: Health and Welfare Survey: 51.3	Pap smear, VIA	Health and Welfare Survey: 46.3%	5
Polrit et al. (2015) [65]	Thailand	Case-control/interview with structured questionnaire	452	Mean (SD) Case: 45.8 (7.8) Controls: 44.8 (9.8)	Pap smear, VIA	NA	5
Srisuwan et al. (2015) [60]	Thailand	Cross-sectional/survey questionnaire and interview	128 village health volunteers/ 10 patients	Mean: Village health volunteer: 46.8	Pap smear	Unspecified	3
Visanuyothin et al. (2015) [52]	Thailand	Cross-sectional/survey questionnaire	595	Distribution: 30–39: 154 (25.9%) 40–49: 219 (36.8%) 50–60: 222 (37.3%)	Pap smear	Within 5-year interval: 65.4%	5
Chaowawanit et al. (2016) [58]	Thailand	Cross-sectional/survey questionnaire	4339	Mean (SD): 46.6 (9.9)	Unspecified	65.3% Within 5-year interval: 42.8%	5
Kittisiam et al. (2016) [67]	Thailand	Cross-sectional/survey questionnaire	2810	Mean (SD): 46.9 (9.9)	Self-HPV test	Unspecified	5
Mongsawaeng et al. (2016) [57]	Thailand	Cross-sectional/survey questionnaire	265	Distribution: 30–40: 63 (23.8%) 41–50: 113 (42.6%) 51–60: 89 (33.6%)	Pap smear	89.8%	5
Chongthawonsatid et al. (2017) [70]	Thailand	Cross-sectional/interview with structured questionnaire	15,074,126	Distribution: 30–39: 5,561,306 (36.9%) 40–49: 5,487,316 (36.4%) 50–59: 4,025,504 (26.7%)	Pap smear	68.4%	5
Gottschlich et al. (2019) [48]	Thailand	Cross-sectional/survey questionnaire	267	Mean (SD): 50.4 (5.8)	Pap smear	82.0%	5
Bunkarn et al. (2020) [63]	Thailand	Prospective quasi-experimental/survey questionnaire	130	Mean (SD): 44.5 (8.3)	Unspecified	Unspecified	4
Songsiriphan et al. (2020) [69]	Thailand	Cross-sectional/survey questionnaire	300	Mean (SD): 45.0 (9.5)	Pap smear	89.3%	4
Vo et al. (2018) [106]	Vietnam	Qualitative/interview	86	Distribution: 20–24: 15 (17.4%) 25–29: 12 (14.0%) 30–34: 32 (37.2%) 35–39: 27 (31.4%)	Pap smear, VIA	3.1%	4
Hoang et al. (2018) [107]	Vietnam	Mixed method/survey questionnaire and interview	130 (113 females)	Mean (SD): Residents: 21 (2.0) Migrants: 22 (1.7)	Pap smear	7.1%	4

HPV: Human papilloma virus; MMAT: Mixed Methods Appraisal Tool; VIA: Visual inspection with acetic acid.

## Data Availability

No new data were created or analyzed in this study. Data sharing is not applicable to this article.

## Supplementary Materials

The following are available online at https://www.mdpi.com/article/10.3390/ijerph18094586/s1, Table S1: Search strategy in each database. Table S2: Quality assessment of included studies using the Mixed Methods Appraisal Tool. Table S3: Barriers to cervical cancer screening in Southeast Asia. Table S4: Facilitators of cervical cancer screening in Southeast Asia. Figure S1: Factors described as barriers to and facilitators of cervical cancer screening in Southeast Asia, according to country and publication numbers.
