# Supplementary material for: Barriers to and Facilitators of Cervical Cancer Screening among Women in Southeast Asia: A Systematic Review
Source: Int J Environ Res Public Health. 2021 Apr 26;18(9):4586. doi: 10.3390/ijerph18094586 (PMC8123618; doi:10.3390/ijerph18094586)
Supplement: Supplementary file 1 [file ijerph-18-04586-s001.zip › Table S1 Search strategy.docx]

**Table S1.** Search strategy in each database

| **Pubmed** | | | | |
| --- | --- | --- | --- | --- |
| **SN** | **Search concept** | **Sub-category** | **Search terms** | **Search hits** |
| 1 | Country of interest | Individual country | **Key words**  Brunei*[tiab] OR Cambodia*[tiab] OR “khmer”[tiab] OR “Laos”[tiab] OR “Lao”[tiab] OR “Laotian”[tiab] OR Myanmar*[tiab] OR “Burma”[tiab] OR “Burmese”[tiab] OR Malay*[tiab] OR “Thailand”[tiab] OR “thai”[tiab] OR Vietnam*[tiab] OR “viet nam”[tiab] OR “East Timor”[tiab] OR “Timor Leste”[tiab] OR “timor-leste”[tiab] OR “Timorese”[tiab] OR Indonesia*[tiab] OR “Philippines”[tiab] OR Filipino*[tiab] OR Singapore*[tiab]  **Controlled vocabulary**  Brunei[mesh] OR Cambodia[mesh] OR Laos[mesh] OR Myanmar[mesh] OR Burma[mesh] OR Malaysia[mesh] OR Thailand[mesh] OR Vietnam[mesh] OR Timor-Leste[mesh] OR Indonesia[mesh] OR Philippines[mesh] OR Singapore[mesh] | 142,299 |
| 2 |  | Region | **Key words**  “ASEAN”[tiab] OR “south east asia*”[tiab] OR “southeast asia*”[tiab]  **Controlled vocabulary**  “Asia, Southeastern”[mesh] | 107,946 |
| 3 | Cervical cancer screening | Specific cervical cancer screening | **Key words**  “Cervical cancer screen*”[tiab] OR “Cervical cancer prevention”[tiab] OR “HPV test*”[tiab] OR “HPV DNA test*”[tiab] OR “Human papillomavirus test*”[tiab] OR “Human papillomavirus DNA test*”[tiab] OR “Human papillomavirus genotyp*”[tiab] OR “Human papillomavirus DNA genotyp*”[tiab] OR “HPV genotyp*”[tiab] OR “HPV DNA genotyp*”[tiab] OR “Schiller's test”[tiab] OR "conventional cytology"[tiab] OR "liquid based cytology"[tiab] OR “colposcop*”[tiab] OR ((cervical[tiab] OR cervico*[tiab] OR cervix[tiab] OR vagina*[tiab] OR Pap[tiab] OR Papanicolaou[tiab]) AND (test*[tiab] OR smear*[tiab] OR swab*[tiab] OR screen*[tiab] OR scrap*[tiab])) OR (“visual inspection”[tiab] AND (“acetic acid”[tiab] OR "lugol's iodine"[tiab]))  **Controlled vocabulary**  “colposcopy”[mesh] OR “vaginal Smears”[MeSH] OR “Papanicolaou Test”[mesh] OR “Human Papillomavirus DNA Tests”[mesh] | 100,971 |
| 4 | Cervical cancer screening | Disease | **Key words**  (cervical[tiab] OR cervix[tiab] OR cervico*[tiab]) AND (neoplasm*[tiab] OR cancer*[tiab] OR carcinoma*[tiab] OR adenocarcinoma*[tiab] OR neoplas*[tiab] OR dysplasia*[tiab] OR dyskaryos*[tiab])  **Controlled vocabulary**  “Uterine Cervical Dysplasia”[mesh] OR “Cervical Intraepithelial Neoplasia”[mesh] OR “Uterine Cervical Neoplasms”[Mesh] | 22,727 |
| 5 |  | Screening | **Key words**  Screen*[tiab] OR “preventive test*”[tiab] OR “preventive investigation*”[tiab] OR “preventive care”[tiab] OR “preventive effort*” OR “preventive management*” OR “early diagnosis”[tiab]  **Controlled vocabulary**  “Early Diagnosis”[MeSH] OR “Early Detection of Cancer”[Mesh] OR “Mass Screening”[MeSH] |  |
| 6 | Barriers | Barriers, facilitators, difficulty, obstruction | **Key words**  Barrier*[tiab] OR factor*[tiab] OR risk*[tiab] OR “risk factor*”[tiab] OR uncertaint*[tiab] OR mistrust*[tiab] OR obstacle*[tiab] OR hurdle*[tiab] OR difficult*[tiab] OR obstruction*[tiab] OR impediment*[tiab] OR challenge*[tiab] OR confront*[tiab] OR “defy”[tiab] OR “defiance*”[tiab] OR object*[tiab] OR contest*[tiab] OR question*[tiab] OR “Health Services Access*”[tiab] OR “healthcare access*”[tiab] OR hinder*[tiab] OR hindrance*[tiab] OR inhibitor*[tiab] OR roadblock*[tiab] OR block*[tiab] OR pitfall*[tiab] OR “Physician Patient Relation*”[tiab] OR “Patient physician Relation*”[tiab] OR “doctor patient relation*”[tiab] OR “patient doctor relation*”[tiab] OR “Communication Barrier*”[tiab] OR “language barrier*”[tiab] OR “procrastinat*”[tiab] OR enabler*[tiab] OR promoter*[tiab] OR facilitator*[tiab] OR cost*[tiab] OR afford*[tiab] OR transport*[tiab] OR financ*[tiab] OR “lack of time”[tiab] OR inconvenien*[tiab] OR predictor*[tiab] OR determinant*[tiab]  **Controlled vocabulary**  risk[MeSH] OR “risk factors”[MeSH] OR uncertainty[MeSH] OR “Health Services Accessibility”[mesh] OR “Physician-Patient Relations”[MeSH] OR “Communication Barriers”[MeSH] OR “Costs and Cost Analysis”[mesh] OR “Socioeconomic Factors”[mesh] OR “transportation”[mesh] OR poverty[mesh] OR “time factors”[mesh] OR “Epidemiologic Factors”[mesh] OR “Social Determinants of Health”[mesh] | 14,912,427 |
| 7 | Perception, beliefs, attitudes, knowledge | Perception, beliefs, attitudes, knowledge, behavior | **Key words**  “Prejudice*”[tiab] OR “self-conscience*”[tiab] OR issue*[tiab] OR attitude*[tiab] OR “belie*”[tiab] OR “health behavior*”[tiab] OR “health behaviour*”[tiab] OR “perception*”[tiab] OR “aware*”[tiab] OR “feeling*”[tiab] OR “opinion*”[tiab] OR “thought*”[tiab] OR “experience*”[tiab] OR “knowledge”[tiab] OR “motivat*”[tiab] OR “understand*”[tiab] OR embarrass*[tiab] OR “fear*”[tiab] OR “accept*”[tiab] OR “health literacy”[tiab] OR cultur*[tiab] OR “shame”[tiab]  **Controlled vocabulary**  “prejudice”[MeSH] OR “attitude”[MeSH] OR “Attitude of Health Personnel”[MeSH] OR “Attitude to Health” [MeSH] OR “Health Knowledge, Attitudes, Practice”[MeSH] OR “Health Behavior”[mesh] OR fear[mesh] OR embarrassment[mesh] OR shame[mesh] OR “patient acceptance of health care”[mesh] OR “health literacy”[mesh] OR health education[mesh] OR culture[mesh] | 5,974,120 |
| 8 | NA | NA | (1 OR 2) AND (3 OR (4 AND 5)) AND (6 OR 7) | 1080 |
| 9 | NA | NA | 8 AND filters (journal article) | 1067 |

| **Embase** | | | | |
| --- | --- | --- | --- | --- |
| **SN** | **Search concept** | **Sub-category** | **Search terms** | **Search hits** |
| 1 | Country of interest | Individual country | **Key words**  Brunei*:ti,ab OR Cambodia*:ti,ab OR ‘khmer’:ti,ab OR ‘Laos’:ti,ab OR ‘Lao’:ti,ab OR ‘Laotian’:ti,ab OR Myanmar*:ti,ab OR ‘Burma’:ti,ab OR Burma/exp OR ‘Burmese’:ti,ab OR Malay*:ti,ab OR ‘Thailand’:ti,ab OR ‘thai’:ti,ab OR Vietnam*:ti,ab OR ‘viet nam’:ti,ab OR ‘East Timor’:ti,ab OR ‘Timor Leste’:ti,ab OR ‘timor-leste’:ti,ab OR ‘Timorese’:ti,ab OR Indonesia*:ti,ab OR ‘Philippines’:ti,ab OR Filipino*:ti,ab OR Singapore*:ti,ab  **Controlled vocabulary**  ‘Brunei Darussalam’/exp OR Cambodia/exp OR Laos/exp OR Myanmar/exp OR Malaysia/exp OR Thailand/exp OR ‘Viet nam’/exp OR Timor-Leste/exp OR Indonesia/exp OR Philippines/exp OR Singapore/exp | 186,596 |
| 2 |  | Region | **Key words**  ‘ASEAN’:ti,ab OR ‘south east asia*’:ti,ab OR ‘southeast asia*’:ti,ab  **Controlled vocabulary**  ‘southeast asia’/exp | 144,228 |
| 3 | Cervical cancer screening | Specific cervical cancer screening | **Key words**  ‘Cervical cancer screen*’:ti,ab OR ‘Cervical cancer prevention’:ti,ab OR ‘HPV test*’:ti,ab OR ‘HPV DNA test*’:ti,ab OR ‘Human papillomavirus test*’:ti,ab OR ‘Human papillomavirus DNA test*’:ti,ab OR ‘Human papillomavirus genotyp*’:ti,ab OR ‘Human papillomavirus DNA genotyp*’:ti,ab OR ‘HPV genotyp*’:ti,ab OR ‘HPV DNA genotyp*’:ti,ab OR ‘colposcop*’:ti,ab OR ‘conventional cytology’:ti,ab OR ‘liquid based cytology’:ti,ab OR ‘vaginal smear*’:ti,ab OR ((‘visual inspection’:ti,ab) AND (‘acetic acid’:ti,ab OR ‘lugol’:ti,ab)) OR ‘schiller test’:ti,ab OR ((cervical:ti,ab OR cervico*:ti,ab OR cervix:ti,ab OR vagina*:ti,ab OR Pap:ti,ab OR Papanicolaou:ti,ab ) AND (test*:ti,ab OR smear*:ti,ab OR swab*:ti,ab OR screen*:ti,ab OR scrap*:ti,ab))  **Controlled vocabulary**  ‘colposcopy’/exp OR ‘vagina Smear’/exp OR ‘Papanicolaou Test’/exp OR ‘Human Papillomavirus DNA Test’/exp | 146,007 |
| 4 | Cervical cancer screening | Disease | **Key words**  (cervical:ti,ab OR cervix:ti,ab OR cervico*:ti,ab) AND (neoplasm*:ti,ab OR cancer*:ti,ab OR carcinoma*:ti,ab OR adenocarcinoma*:ti,ab OR neoplas*:ti,ab OR dysplasia*:ti,ab OR dyskaryos*:ti,ab)  **Controlled vocabulary**  (‘uterine cervix dysplasia’/exp OR ‘uterine cervix cancer’/exp OR ‘uterine cervix tumor’/exp) | 55,229 |
| 5 |  | Screening | **Key words**  (Screen*:ti,ab OR ‘preventive test*’:ti,ab OR ‘preventive investigation*’:ti,ab OR ‘preventive care’:ti,ab OR ‘preventive effort*’ OR ‘preventive management*’ OR ‘early diagnosis’:ti,ab)  **Controlled vocabulary**  (‘Mass Screening’/exp OR ‘cancer diagnosis’/exp OR ‘Early Diagnosis’/exp) |  |
| 6 | Barriers | Barriers,  Facilitators, difficulty, obstruction | **Key words**  Barrier*:ti,ab OR factor*:ti,ab OR risk*:ti,ab OR ‘risk factor*’:ti,ab OR ‘uncertaint*’:ti,ab OR ‘mistrust*’:ti,ab OR obstacle*:ti,ab OR hurdle*:ti,ab OR ‘difficult*’:ti,ab OR ‘obstruction*’:ti,ab OR ‘impediment*’:ti,ab OR Challenge*:ti,ab OR confront*:ti,ab OR ‘defy’:ti,ab OR ‘defiance*’:ti,ab OR object*:ti,ab OR contest*:ti,ab OR question*:ti,ab OR ‘Health Services Access*’:ti,ab OR ‘healthcare access*’:ti,ab OR hinder*:ti,ab OR hindrance*:ti,ab OR inhibitor*:ti,ab OR roadblock*:ti,ab OR block*:ti,ab OR pitfall*:ti,ab OR ‘doctor patient relation*’:ti,ab OR ‘patient doctor relation*’:ti,ab OR ‘Physician Patient Relation*’:ti,ab OR ‘Patient physician Relation*’:ti,ab OR ‘Communication Barrier*’:ti,ab OR ‘language barrier*’:ti,ab OR ‘procrastinat*’:ti,ab OR ‘enabler*’:ti,ab OR ’promoter*’:ti,ab OR ‘facilitator*’:ti,ab OR ‘cost*’:ti,ab OR ‘afford*’:ti,ab OR transport*:ti,ab OR financ*:ti,ab OR ‘lack of time’:ti,ab OR inconvenien*:ti,ab OR predictor*:ti,ab OR determinant*:ti,ab  **Controlled vocabulary**  risk/exp OR risk factor/exp OR uncertainty/exp OR ‘health care access’/exp OR ‘doctor patient relationship’/exp OR ‘communication barrier’/exp OR 'affordability'/exp OR 'cost'/exp OR 'facilitator'/exp OR Socioeconomics/exp OR poverty/exp OR ‘time factor’/exp OR ‘determinants’/exp OR ‘social determinants of health’/exp OR ‘predictors’/exp OR ‘predictor variable’/exp | 14,660,059 |
| 7 | Perception, beliefs, attitudes, knowledge | Perception, beliefs, attitudes, knowledge, behavior | **Key words**  ‘Prejudice*’:ti,ab OR ‘self-conscience*’:ti,ab OR issue*:ti,ab OR attitude*:ti,ab OR ‘belie*’:ti,ab OR ‘health behavior*’:ti,ab OR ‘health behaviour*’:ti,ab OR ‘perception*’:ti,ab OR ‘aware*’:ti,ab OR ‘feeling*’:ti,ab OR ‘opinion*’:ti,ab OR ‘thought*’:ti,ab OR ‘experience*’:ti,ab OR ‘knowledge’:ti,ab OR ‘motivat*’:ti,ab OR ‘understand*’:ti,ab OR embarrass*:ti,ab OR fear*:ti,ab OR accept*:ti,ab OR ‘health literacy’:ti,ab OR cultur*:ti,ab OR shame:ti,ab  **Controlled vocabulary**  ‘prejudice’/exp OR ‘attitude’/exp OR ‘health personnel attitude’/exp OR ‘Attitude to Health’/exp OR ‘health behavior’/exp OR fear/exp OR embarrassment/exp OR shame/exp OR ‘patient attitude’/exp OR ‘health literacy’/exp OR ‘health education’/exp OR ‘cultural factor’/exp | 7,842,615 |
| 8 | NA | NA | (1 OR 2) AND (3 OR (4 AND 5)) AND (6 OR 7) | 1646 |
| 9 | NA | NA | 8 AND filters (article in press, article) | 1076 |

| **CINAHL** | | | | |
| --- | --- | --- | --- | --- |
| **SN** | **Search concept** | **Sub-category** | **Search terms** | **Search hits** |
| 1 | Country of interest | Individual country | **Key words (title and abstract)**  Brunei* OR Cambodia* OR “khmer” OR “Laos” OR “Lao” OR “Laotian” OR Myanmar* OR “Burma” OR “Burmese” OR Malay* OR “Thailand” OR “thai” OR Vietnam* OR “viet nam” OR “East Timor” OR “Timor Leste” OR “timor-leste” OR “Timorese” OR Indonesia* OR “Philippines” OR Filipino* OR Singapore*  **Controlled vocabulary**  (MH “Brunei”) OR (MH “Cambodia”) OR (MH “Laos”) OR (MH “Myanmar”) OR (MH “Burma”) OR (MH “Malaysia”) OR (MH “Thailand”) OR (MH “Vietnam”) OR (MH “east timor”) OR (MH “Indonesia”) OR (MH “Philippines”) OR (MH “Singapore”) | 42,168 |
| 2 |  | Region | **Key words (title and abstract)**  “ASEAN” OR “south east asia*” OR “southeast asia*”  **Controlled vocabulary**  (MH "Asia, Southeastern+") | 34,842 |
| 3 | Cervical cancer screening | Specific cervical cancer screening | **Key words (title and abstract)**  “Cervical cancer screen*” OR “Cervical cancer prevention” OR “HPV test*” OR “HPV DNA test*” OR “Human papillomavirus test*” OR “Human papillomavirus DNA test*” OR “Human papillomavirus genotyp*” OR “Human papillomavirus DNA genotyp*” OR “HPV genotyp*” OR “HPV DNA genotyp*” OR “Schiller's test” OR "conventional cytology" OR "liquid based cytology" OR “colposcop*” OR ((cervical OR cervico* OR cervix OR vagina* OR Pap OR Papanicolaou) AND (test* OR smear* OR swab* OR screen* OR scrap*)) OR (“visual inspection” AND (“acetic acid” OR "lugol's iodine"))  **Controlled vocabulary**  (MH “colposcopy”) OR (MH "Cervical Smears+") | 24,951 |
| 4 | Cervical cancer screening | Disease | **Key words (title and abstract)**  (cervical OR cervix OR cervico*) AND (neoplasm* OR cancer* OR carcinoma* OR adenocarcinoma* OR neoplas* OR dysplasia* OR dyskaryos*)  **Controlled vocabulary**  (MH “Cervix Dysplasia”) OR (MH "Cervical Intraepithelial Neoplasia+") OR (MH "Cervix Neoplasms+") | 8,422 |
| 5 |  | Screening | **Key words (title and abstract)**  Screen* OR “preventive test*” OR “preventive investigation*” OR “preventive care” OR “preventive effort*” OR “preventive management*” OR “early diagnosis”  **Controlled vocabulary**  (MH "Early Diagnosis+") OR (MH “Early Detection of Cancer” OR (MH “Health Screening+”) |  |
| 6 | Barriers | Barriers, facilitators, difficulty, obstruction | **Key words (title and abstract)**  Barrier* OR factor* OR risk* OR “risk factor*” OR uncertaint* OR mistrust* OR obstacle* OR hurdle* OR difficult* OR obstruction* OR impediment* OR challenge* OR confront* OR “defy” OR “defiance*” OR object* OR contest* OR question* OR “Health Services Access*” OR “healthcare access*” OR hinder* OR hindrance* OR inhibitor* OR roadblock* OR block* OR pitfall* OR “Physician Patient Relation*” OR “Patient physician Relation*” OR “doctor patient relation*” OR “patient doctor relation*” OR “Communication Barrier*” OR “language barrier*” OR “procrastinat*” OR enabler* OR promoter* OR facilitator* OR cost* OR afford* OR transport* OR financ* OR “lack of time” OR inconvenien* OR predictor* OR determinant*  **Controlled vocabulary**  (MH “risk factors+”) OR (MH “uncertainty”) OR (MH "Health Services Accessibility+") OR (MH "Physician-Patient Relations") OR (MH "Communication Barriers+") OR (MH "Costs and Cost Analysis+") OR (MH "Socioeconomic Factors+") OR (MH “transportation+”) OR (MH “poverty+”) OR (MH “time factors”) OR (MH "Social Determinants of Health") | 2,804,843 |
| 7 | Perception, beliefs, attitudes, knowledge | Perception, beliefs, attitudes, knowledge, behavior | **Key words (title and abstract)**  “Prejudice*” OR “self-conscience*” OR issue* OR attitude* OR “belie*” OR “health behavior*” OR “health behaviour*” OR “perception*” OR “aware*” OR “feeling*” OR “opinion*” OR “thought*” OR “experience*” OR “knowledge” OR “motivat*” OR “understand*” OR embarrass* OR “fear*” OR “accept*” OR “health literacy” OR cultur* OR “shame”  **Controlled vocabulary**  (MH “prejudice+”) OR (MH “attitude+”) OR (MH “Attitude of Health Personnel+”) OR (MH “Attitude to Health+”) OR (MH “Health Behavior+”) OR (MH "Health Knowledge (Iowa NOC)+") OR (MH "Health Behavior (Iowa NOC)+") OR (MH "Health Knowledge") OR (MH "Health Knowledge and Behavior (Iowa NOC)+") OR (MH "Knowledge: Health Resources (Iowa NOC)") OR (MH "Knowledge: Health Behaviors (Iowa NOC)") OR (MH "Health Promoting Behavior (Iowa NOC)") OR (MH "Health Seeking Behaviors (NANDA)+") OR (MH "Embarrassment") OR (MH “shame+”) OR (MH “health literacy”) OR (MH “health education+” OR (MH “culture+”) OR (MH "Fear (Saba CCC)") OR (MH "Fear (NANDA)") OR (MH "Fear+") | 1,833,656 |
| 8 | NA | NA | (1 OR 2) AND (3 OR (4 AND 5)) AND (6 OR 7) | 341 |
| 9 | NA | NA | 8 AND filters (Publication type: journal article, Geographic subset: Asia) | 17 |

| **PsycInfo** | | | | |
| --- | --- | --- | --- | --- |
| **SN** | **Search concept** | **Sub-category** | **Search terms** | **Search hits** |
| 1 | Country of interest | Individual country | **Key words**  (Brunei* or Cambodia* or khmer or Laos or Lao or Laotian or Myanmar* or Burma or Burmese or Malaysia* or Thailand or thai or Vietnam* or viet nam or East Timor or Timor Leste or timor-leste or Timorese or Indonesia* or Philippines or Filipino* or Singapore*).ti,ab.  **Controlled vocabulary**  exp Brunei/ or exp Cambodia/ or exp Laos/ or exp Burma/ or exp Myanmar/ or exp Thailand/ or exp Timor-Leste/ or exp Timor-Leste/ or exp Indonesia/ or exp Philippines/ or exp Vietnam/ or exp Malaysia/ or exp Singapore/ | 27,264 |
| 2 |  | Region | **Key words**  (ASEAN or south east asia* or southeast asia*).ti,ab.  **Controlled vocabulary**  exp southeast asia/ or exp south east asia/ or exp Southeast Asian Cultural Groups/ | 3,529 |
| 3 | Cervical cancer screening | Specific cervical cancer screening terms | **Key words**  (Cervical cancer screen* OR Cervical cancer prevention OR HPV test* OR HPV DNA test* OR Human papillomavirus test* OR Human papillomavirus DNA test* OR Human papillomavirus genotyp* OR Human papillomavirus DNA genotyp* OR HPV genotyp* OR HPV DNA genotyp* OR Schiller's test OR conventional cytology OR liquid based cytology OR colposcop* OR ((cervical OR cervico* OR cervix OR vagina* OR Pap OR Papanicolaou) AND (test* OR smear* OR swab* OR screen* OR scrap*)) OR (visual inspection AND (acetic acid OR lugol's iodine))).ti,ab. | 5,041 |
| 4 |  | Disease | **Key words**  ((cervical OR cervix OR cervico*) AND (neoplasm* OR cancer* OR carcinoma* OR adenocarcinoma* OR neoplas* OR dysplasia* OR dyskaryos*)).ti,ab.  **Controlled vocabulary**  ((exp Uterine Cervical Neoplasms/ OR exp Uterine Cervical Dysplasia/ OR exp Cervical Intraepithelial Neoplasia/) or (exp cervix/ AND exp neoplasms/)) | 1,589 |
| 5 |  | Screening | **Key words**  (Screen* OR preventive test* OR preventive investigation* OR preventive care OR preventive effort* OR preventive management* OR early diagnosis).ti,ab.  **Controlled vocabulary**  (exp Mass Screening/ OR exp Early Diagnosis/ OR exp Early Detection of Cancer/ or exp health screening/ or exp cancer screening/) |  |
| 6 | Barriers | Barriers, difficulty, obstruction | **Key words**  (Barrier* OR factor* OR risk* OR risk factor* OR uncertaint* OR mistrust* OR obstacle* OR hurdle* OR difficult* OR obstruction* OR impediment* OR challenge* OR confront* OR defy OR defiance* OR object* OR contest* OR question* OR Health Services Access* OR healthcare access* OR hinder* OR hindrance* OR inhibitor* OR roadblock* OR block* OR pitfall* OR Physician Patient Relation* OR Patient physician Relation* OR doctor patient relation* OR patient doctor relation* OR Communication Barrier* OR language barrier* OR procrastinat* OR enabler* OR promoter* OR facilitator* OR cost* OR afford* OR transport* OR financ* OR lack of time OR inconvenien* OR predictor* OR determinant*).ti,ab.  **Controlled vocabulary**  exp risk/ or exp risk factors/ or exp uncertainty/ or exp Physician Patient Relations/ or exp Communication Barriers/ or exp Health Care Access/ or exp Socioeconomic status/ or exp Sociocultural status/ or exp transportation/ or exp poverty/ or exp time/ or exp Demographic Characteristics/ | 2412211 |
| 7 |  | Perception, beliefs, attitudes, knowledge, behavior | **Key words**  (Prejudice* OR self-conscience* OR issue* OR attitude* OR belie* OR health behavior* OR health behaviour* OR perception* OR aware* OR feeling* OR opinion* OR thought* OR experience* OR knowledge OR motivat* OR understand* OR embarrass* OR fear* OR accept* OR health literacy OR cultur* OR shame).ti,ab.  **Controlled vocabulary**  exp prejudice/ OR exp attitudes/ OR exp Health Personnel Attitudes/ OR exp health Attitudes/ OR exp Health Knowledge/ OR exp health behaviour/ OR exp Health Behavior/ OR exp perception/ or exp fear/ or exp embarrassment/ or exp shame/ or exp health education/ | 2,620,507 |
| 8 | NA | NA | (1 OR 2) AND (3 OR (4 AND 5)) AND (6 OR 7) | 110 |
| 9 | NA | NA | 8 AND filters (publication type: journal, peer reviewed journal, Document type: journal article) | 97 |

| **SCOPUS** | | | | |
| --- | --- | --- | --- | --- |
| **SN** | **Search concept** | **Sub-category** | **Search terms** | **Search hits** |
| 1 | Country of interest | Individual country | **Key words**  TITLE-ABS(Brunei* OR Cambodia* OR “khmer” OR “Laos” OR “Lao” OR “Laotian” OR Myanmar* OR “Burma” OR “Burmese” OR Malay* OR “Thailand” OR “thai” OR Vietnam* OR “viet nam” OR “East Timor” OR “Timor Leste” OR “timor-leste” OR “Timorese” OR Indonesia* OR “Philippines” OR Filipino* OR Singapore*) | 567,022 |
| 2 |  | Region | **Key words**  TITLE-ABS(“ASEAN” OR “south east asia*” OR “southeast asia*”) | 55,827 |
| 3 | Cervical cancer screening | Specific cervical cancer screening | **Key words**  TITLE-ABS(“Cervical cancer screen*” OR “Cervical cancer prevention” OR “HPV test*” OR “HPV DNA test*” OR “Human papillomavirus test*” OR “Human papillomavirus DNA test*” OR “Human papillomavirus genotyp*” OR “Human papillomavirus DNA genotyp*” OR “HPV genotyp*” OR “HPV DNA genotyp*” OR “Schiller's test” OR "conventional cytology" OR "liquid based cytology" OR “colposcop*” OR ((cervical OR cervico* OR cervix OR vagina* OR Pap OR Papanicolaou) AND (test* OR smear* OR swab* OR screen* OR scrap*)) OR (“visual inspection” AND (“acetic acid” OR "lugol's iodine"))) | 109,324 |
| 4 | Cervical cancer screening | Disease | **Key words**  TITLE-ABS((cervical OR cervix OR cervico*) AND (neoplasm* OR cancer* OR carcinoma* OR adenocarcinoma* OR neoplas* OR dysplasia* OR dyskaryos*)) | 21,592 |
| 5 |  | Screening | **Key words**  TITLE-ABS(Screen* OR “preventive test*” OR “preventive investigation*” OR “preventive care” OR “preventive effort*” OR “preventive management*” OR “early diagnosis”) |  |
| 6 | Barriers | Barriers, facilitators, difficulty, obstruction | **Key words**  TITLE-ABS(Barrier* OR factor* OR risk* OR “risk factor*” OR uncertaint* OR mistrust* OR obstacle* OR hurdle* OR difficult* OR obstruction* OR impediment* OR challenge* OR confront* OR “defy” OR “defiance*” OR object* OR contest* OR question* OR “Health Services Access*” OR “healthcare access*” OR hinder* OR hindrance* OR inhibitor* OR roadblock* OR block* OR pitfall* OR “Physician Patient Relation*” OR “Patient physician Relation*” OR “doctor patient relation*” OR “patient doctor relation*” OR “Communication Barrier*” OR “language barrier*” OR “procrastinat*” OR enabler* OR promoter* OR facilitator* OR cost* OR afford* OR transport* OR financ* OR “lack of time” OR inconvenien* OR predictor* OR determinant*) | 23,467,014 |
| 7 | Perception, beliefs, attitudes, knowledge | Perception, beliefs, attitudes, knowledge, behaviour | **Key words**  TITLE-ABS(“Prejudice*” OR “self-conscience*” OR issue* OR attitude* OR “belie*” OR “health behavior*” OR “health behaviour*” OR “perception*” OR “aware*” OR “feeling*” OR “opinion*” OR “thought*” OR “experience*” OR “knowledge” OR “motivat*” OR “understand*” OR embarrass* OR “fear*” OR “accept*” OR “health literacy” OR cultur* OR “shame”) | 13,345,545 |
| 8 | NA | NA | (1 OR 2) AND (3 OR (4 AND 5)) AND (6 OR 7) | 1,143 |
| 9 | NA | NA | 8 AND filters  Country: Malaysia, Thailand, Indonesia, Singapore, Viet Nam, Philippines, Cambodia, Laos, Brunei Darussalam, Myanmar, Undefined  Document type: articles  Source type: journal | 768 |
