# Supplementary material for: Barriers to and Facilitators of Cervical Cancer Screening among Women in Southeast Asia: A Systematic Review
Source: Int J Environ Res Public Health. 2021 Apr 26;18(9):4586. doi: 10.3390/ijerph18094586 (PMC8123618; doi:10.3390/ijerph18094586)
Supplement: Supplementary file 1 [file ijerph-18-04586-s001.zip › Table S2 MMAT.docx]

**Table S2**. Quality assessment of included studies using the Mixed Methods Appraisal Tool

| **Author (year)** | **Criteria from the Mixed Methods Appraisal Tool*** | | | | | | | | | | | | | | | | | | | |
| --- | --- | --- | --- | --- | --- | --- | --- | --- | --- | --- | --- | --- | --- | --- | --- | --- | --- | --- | --- | --- |
|  | **Qualitative studies** | | | | | **Non-randomized studies** | | | | | **Quantitative descriptive studies** | | | | | **Mixed methods studies** | | | | |
|  | **1.1.** | **1.2** | **1.3** | **1.4** | **1.5** | **3.1** | **3.2** | **3.3** | **3.4** | **3.5** | **4.1** | **4.2** | **4.3** | **4.4** | **4.5** | **5.1** | **5.2** | **5.3** | **5.4** | **5.5** |
| Seow et al. (1994) |  |  |  |  |  | Yes | Yes | Yes | Yes | Yes |  |  |  |  |  |  |  |  |  |  |
| Seow et al. (1995) |  |  |  |  |  | Yes | Yes | Yes | Yes | Yes |  |  |  |  |  |  |  |  |  |  |
| Seow et al. (2000) |  |  |  |  |  | Yes | Yes | Yes | Yes | Yes |  |  |  |  |  |  |  |  |  |  |
| Lee et al. (2002) |  |  |  |  |  | Yes | Yes | Yes | Yes | Yes |  |  |  |  |  |  |  |  |  |  |
| Boonmongkon et al. (2002) |  |  |  |  |  |  |  |  |  |  | Can't tell | Can't tell | Yes | Can't tell | Yes |  |  |  |  |  |
| Chee et al. (2003) |  |  |  |  |  | No | Yes | Yes | Yes | Yes |  |  |  |  |  |  |  |  |  |  |
| Chee et al. (2003) |  |  |  |  |  | Can't tell | No | Yes | No | Yes |  |  |  |  |  |  |  |  |  |  |
| Kritpetcharat et al. (2003) |  |  |  |  |  |  |  |  |  |  | Yes | Can't tell | Yes | Can't tell | Yes |  |  |  |  |  |
| Chalapati et al. (2007) |  |  |  |  |  | Yes | Yes | Yes | Yes | Yes |  |  |  |  |  |  |  |  |  |  |
| Moy et al. (2007) |  |  |  |  |  | No | Yes | Yes | Yes | Yes |  |  |  |  |  |  |  |  |  |  |
| Asmani et al. (2007) |  |  |  |  |  | Yes | Yes | Yes | No | Yes |  |  |  |  |  |  |  |  |  |  |
| Boonpongmanee et al. (2007) |  |  |  |  |  | No | Yes | Yes | No | Yes |  |  |  |  |  |  |  |  |  |  |
| Wong et al. (2008) | Yes | Yes | Can't tell | Yes | Yes |  |  |  |  |  |  |  |  |  |  |  |  |  |  |  |
| Othman et al. (2009) |  |  |  |  |  | Can't tell | No | Yes | Can’t tell | Yes |  |  |  |  |  |  |  |  |  |  |
| Wong et al. (2009) | Yes | Yes | Yes | Yes | Yes |  |  |  |  |  |  |  |  |  |  |  |  |  |  |  |
| Kietpeerakool et al. (2009) |  |  |  |  |  | Can't tell | Yes | Yes | No | Yes |  |  |  |  |  |  |  |  |  |  |
| Dunn et al. (2010) |  |  |  |  |  | Yes | No | Yes | Yes | Yes |  |  |  |  |  |  |  |  |  |  |
| Abdullah et al. (2010) | Yes | Yes | Yes | Yes | Yes |  |  |  |  |  |  |  |  |  |  |  |  |  |  |  |
| Oon et al. (2010) | Yes | Yes | Yes | Yes | Yes |  |  |  |  |  |  |  |  |  |  |  |  |  |  |  |
| Al-Naggar et al. (2010) |  |  |  |  |  | Yes | No | Yes | No | Yes |  |  |  |  |  |  |  |  |  |  |
| Oranratanaphan et al. (2010) |  |  |  |  |  |  |  |  |  |  | Can't tell | Can't tell | Yes | Can't tell | Yes |  |  |  |  |  |
| Wee et al. (2010) |  |  |  |  |  | No | Yes | Yes | Yes | Yes |  |  |  |  |  |  |  |  |  |  |
| Al-Naggar et al. (2010) | Yes | Yes | Yes | Yes | Yes |  |  |  |  |  |  |  |  |  |  |  |  |  |  |  |
| Phongsavan et al. (2010) |  |  |  |  |  |  |  |  |  |  | Yes | Yes | Yes | Yes | Yes |  |  |  |  |  |
| Srisakul et al. (2011) |  |  |  |  |  | Can't tell | Can't tell | Yes | Yes | Yes |  |  |  |  |  |  |  |  |  |  |
| Abdullah et al. (2011) |  |  |  |  |  | Yes | Yes | Yes | Yes | Yes |  |  |  |  |  |  |  |  |  |  |
| Thanapprapasr et al. (2012) |  |  |  |  |  | Yes | Yes | Yes | No | Yes |  |  |  |  |  |  |  |  |  |  |
| Chesun et al. (2012) |  |  |  |  |  | Yes | Can't tell | Yes | Yes | Yes |  |  |  |  |  |  |  |  |  |  |
| Kim et al. (2012) | Yes | Yes | Yes | Yes | Yes |  |  |  |  |  |  |  |  |  |  |  |  |  |  |  |
| Al-Naggar et al. (2012) |  |  |  |  |  | No | No | Yes | Yes | Yes |  |  |  |  |  |  |  |  |  |  |
| Wee et al. (2012) |  |  |  |  |  | No | Yes | Yes | Yes | Yes |  |  |  |  |  |  |  |  |  |  |
| Gan et al. (2013) |  |  |  |  |  | Yes | Yes | Yes | Yes | Yes |  |  |  |  |  |  |  |  |  |  |
| Wong et al. (2013) |  |  |  |  |  | Yes | No | Yes | Yes | Yes |  |  |  |  |  |  |  |  |  |  |
| Baskaran et al. (2013) |  |  |  |  |  | Can't tell | Yes | Yes | No | Yes |  |  |  |  |  |  |  |  |  |  |
| Aziz et al. (2013) |  |  |  |  |  | Yes | No | Yes | Yes | Yes |  |  |  |  |  |  |  |  |  |  |
| Shea et al. (2013) |  |  |  |  |  | No | Yes | Yes | No | Yes |  |  |  |  |  |  |  |  |  |  |
| Oranratanaphan et al. (2014) |  |  |  |  |  |  |  |  |  |  | Can't tell | No | Yes | Can't tell | Yes |  |  |  |  |  |
| Wongwatcharanukul et al. (2014) |  |  |  |  |  | Yes | Can't tell | Yes | Yes | Yes |  |  |  |  |  |  |  |  |  |  |
| Budkaew et al. (2014) |  |  |  |  |  | Can't tell | Can't tell | Yes | Yes | Yes |  |  |  |  |  |  |  |  |  |  |
| Sichanh et al. (2014) |  |  |  |  |  |  |  |  |  |  | Yes | Yes | Yes | Yes | Yes |  |  |  |  |  |
| Chirayil et al. (2014) |  |  |  |  |  | No | Yes | Yes | No | Yes |  |  |  |  |  |  |  |  |  |  |
| Visanuyothin et al. (2015) |  |  |  |  |  | Yes | Yes | Yes | Yes | Yes |  |  |  |  |  |  |  |  |  |  |
| Nandar et al. (2015) |  |  |  |  |  | Yes | Yes | Yes | Yes | Yes |  |  |  |  |  |  |  |  |  |  |
| Tay et al. (2015) |  |  |  |  |  | No | No | Yes | Yes | Yes |  |  |  |  |  |  |  |  |  |  |
| Wong et al. (2015) |  |  |  |  |  | Yes | Yes | Yes | Yes | Yes |  |  |  |  |  |  |  |  |  |  |
| Azrai et al. (2015) |  |  |  |  |  | No | No | Yes | Yes | Yes |  |  |  |  |  |  |  |  |  |  |
| Srisuwan et al. (2015) |  |  |  |  |  | Can't tell | Yes | Yes | No | Yes |  |  |  |  |  |  |  |  |  |  |
| Mukem et al. (2015) |  |  |  |  |  | Yes | Yes | Yes | Yes | Yes |  |  |  |  |  |  |  |  |  |  |
| Polrit et al. (2015) |  |  |  |  |  | Yes | Yes | Yes | Yes | Yes |  |  |  |  |  |  |  |  |  |  |
| Ma'som et al. (2016) |  |  |  |  |  | Can't tell | Yes | Yes | Yes | Yes |  |  |  |  |  |  |  |  |  |  |
| Abdullah et al. (2016) |  |  |  |  |  | Yes | Yes | Yes | Yes | Yes |  |  |  |  |  |  |  |  |  |  |
| Danial (et al. (2016) |  |  |  |  |  | Can't tell | Yes | Yes | Yes | Yes |  |  |  |  |  |  |  |  |  |  |
| Kittisiam et al. (2016) |  |  |  |  |  | Yes | Yes | Yes | Yes | Yes |  |  |  |  |  |  |  |  |  |  |
| Mongsawaeng et al. (2016) |  |  |  |  |  |  |  |  |  |  | Yes | Yes | Yes | Yes | Yes |  |  |  |  |  |
| Chaowawanit et al. (2016) |  |  |  |  |  | Yes | Yes | Yes | Yes | Yes |  |  |  |  |  |  |  |  |  |  |
| Anggraeni et al. (2020) |  |  |  |  |  | Can't tell | Yes | Yes | Yes | Yes |  |  |  |  |  |  |  |  |  |  |
| Wee et al. (2016) | Yes | Yes | Yes | Yes | Yes | Yes | Yes | Yes | Yes | Yes |  |  |  |  |  | Yes | Yes | Yes | Can't tell | Yes |
| Wee et al. (2016) | Yes | Yes | Yes | Yes | Yes |  |  |  |  |  |  |  |  |  |  |  |  |  |  |  |
| Sidabutar et al. (2017) |  |  |  |  |  | Can't tell | Yes | Can't tell | Can't tell | Yes |  |  |  |  |  |  |  |  |  |  |
| Nurhasanah et al. (2017) |  |  |  |  |  | Can't tell | Can't tell | Yes | Yes | Yes |  |  |  |  |  |  |  |  |  |  |
| Wee et al. (2017) | Yes | Yes | Yes | Yes | Yes | Yes | Yes | Yes | Yes | Yes |  |  |  |  |  | Yes | Yes | Yes | Yes | Yes |
| Indra et al. (2017) |  |  |  |  |  | Yes | Yes | Yes | No | Yes |  |  |  |  |  |  |  |  |  |  |
| Razi et al. (2017) |  |  |  |  |  | No | Yes | Yes | Yes | Yes |  |  |  |  |  |  |  |  |  |  |
| Wakhidah et al. (2017) |  |  |  |  |  | Can't tell | Yes | Yes | Yes | Yes |  |  |  |  |  |  |  |  |  |  |
| Rubini et al. (2018) |  |  |  |  |  |  |  |  |  |  | Yes | Can't tell | Yes | Yes | Yes |  |  |  |  |  |
| Saptowati et al. (2018) |  |  |  |  |  | Can't tell | Can't tell | Yes | Yes | Yes |  |  |  |  |  |  |  |  |  |  |
| Anwar et al. (2018) |  |  |  |  |  | Yes | Yes | Yes | Yes | Yes |  |  |  |  |  |  |  |  |  |  |
| Rahmawati et al. (2018) |  |  |  |  |  | Yes | Can't tell | Yes | Yes | Yes |  |  |  |  |  |  |  |  |  |  |
| Yeo et al. (2018) |  |  |  |  |  | No | Yes | Yes | Yes | Yes |  |  |  |  |  |  |  |  |  |  |
| Nwabichie et al. (2018) |  |  |  |  |  | Yes | Yes | Yes | Yes | Yes |  |  |  |  |  |  |  |  |  |  |
| Sidabutar et al. (2018) |  |  |  |  |  | Can't tell | Yes | Yes | Yes | Yes |  |  |  |  |  |  |  |  |  |  |
| Abdullah et al. (2018) |  |  |  |  |  | No | Yes | Yes | Yes | Yes |  |  |  |  |  |  |  |  |  |  |
| Hando et al. (2018) |  |  |  |  |  | No | Yes | Yes | No | Yes |  |  |  |  |  |  |  |  |  |  |
| Vo et al. (2018) | Yes | Yes | Yes | No | Yes |  |  |  |  |  |  |  |  |  |  |  |  |  |  |  |
| Touch et al. (2018) |  |  |  |  |  | Yes | Yes | Yes | Yes | Yes |  |  |  |  |  |  |  |  |  |  |
| Yunus et al. (2018) |  |  |  |  |  | Can't tell | Yes | Yes | Yes | Yes |  |  |  |  |  |  |  |  |  |  |
| Aprina et al. (2018) |  |  |  |  |  | Can't tell | Can't tell | Yes | Yes | Yes |  |  |  |  |  |  |  |  |  |  |
| Sundraraj et al. (2018) |  |  |  |  |  | Can't tell | No | Yes | No | Yes |  |  |  |  |  |  |  |  |  |  |
| Winarti et al. (2018) |  |  |  |  |  | Can't tell | Yes | Can't tell | Yes | Yes |  |  |  |  |  |  |  |  |  |  |
| Hoang et al. (2018) | Yes | Can't tell | Yes | Yes | Yes |  |  |  |  |  | Yes | Yes | Yes | Can't tell | Yes | Yes | Yes | Yes | Yes | Yes |
| Spagnoletti et al. (2019) | Yes | Yes | Yes | No | Yes |  |  |  |  |  |  |  |  |  |  |  |  |  |  |  |
| Sutarti et al. (2018) |  |  |  |  |  | Yes | No | Can't tell | Yes | Yes |  |  |  |  |  |  |  |  |  |  |
| Gottschlich et al. (2019) |  |  |  |  |  | Yes | Yes | Yes | Yes | Yes |  |  |  |  |  |  |  |  |  |  |
| Romli et al. (2019) |  |  |  |  |  | Yes | Yes | Yes | Yes | Yes |  |  |  |  |  |  |  |  |  |  |
| Siraj et al. (2019) |  |  |  |  |  | Can't tell | Yes | Yes | Yes | Yes |  |  |  |  |  |  |  |  |  |  |
| Baharum et al. (2020) |  |  |  |  |  | Yes | Yes | Yes | Yes | Yes |  |  |  |  |  |  |  |  |  |  |
| Muhith et al. (2020) |  |  |  |  |  | Can’t tell | Yes | Can't tell | No | Yes |  |  |  |  |  |  |  |  |  |  |
| Ting et al. (2020) |  |  |  |  |  | Yes | Yes | Yes | No | Yes |  |  |  |  |  |  |  |  |  |  |
| Widayanti et al. (2020) |  |  |  |  |  | Can't tell | Can't tell | Yes | No | Yes |  |  |  |  |  |  |  |  |  |  |
| Suhaimi et al. (2020) |  |  |  |  |  | Yes | Yes | Yes | Yes | Yes |  |  |  |  |  |  |  |  |  |  |
| Bunkarn et al. (2020) |  |  |  |  |  | Can't tell | Yes | Yes | Yes | Yes |  |  |  |  |  |  |  |  |  |  |
| Chongthawonsatid et al. (2017) |  |  |  |  |  | Yes | Yes | Yes | Yes | Yes |  |  |  |  |  |  |  |  |  |  |
| Songsiriphan et al. (2020) |  |  |  |  |  | Can't tell | Yes | Yes | Yes | Yes |  |  |  |  |  |  |  |  |  |  |

* Only applicable fields based on study design are filled for each study
