# Supplementary material for: Barriers to and Facilitators of Cervical Cancer Screening among Women in Southeast Asia: A Systematic Review
Source: Int J Environ Res Public Health. 2021 Apr 26;18(9):4586. doi: 10.3390/ijerph18094586 (PMC8123618; doi:10.3390/ijerph18094586)
Supplement: Supplementary file 1 [file ijerph-18-04586-s001.zip › Table S3 Barriers to screening.docx]

**Table S3.** Barriers to cervical cancer screening in Southeast Asia

| **Categories** | **Barriers** | **Brunei  (n = 1)** | **Indonesia  (n = 7)** | **Laos  (n = 3)** | **Malaysia  (n = 21)** | **Singapore  (n = 10)** | **Thailand  (n = 19)** | **Vietnam  (n = 2)** | **Total number of studies** | **Total number of countries** |
| --- | --- | --- | --- | --- | --- | --- | --- | --- | --- | --- |
| **Demographics**  **(n = 18)** | Age | 1 | - | - | 3 | 1 | 4 | - | 9 | 4 |
|  | Marital status (single) | - | - | - | 1 | - | 3 | - | 4 | 2 |
|  | Ethnicity | - | - | - | 1 | 2 | - | - | 3 | 2 |
|  | Comorbidities | - | - | - | 1 | - | - | - | 1 | 1 |
|  | No family history of cancer | - | - | - | 1 | - | - | - | 1 | 1 |
|  | Religion | - | - | - | - | - | 4 | 1 | 5 | 2 |
|  | No/low children | - | - | 1 | - | - | 2 | - | 3 | 2 |
|  | At least 1 factor within category | ✓ | - | ✓ | ✓ | ✓ | ✓ | ✓ | - | 6 |
| **Socio-economic**  **(n = 8)** | Low education | - | - | - | 1 | - | - | - | 1 | 1 |
|  | Housing type (rental) | - | - | - | - | 1 | - | - | 1 | 1 |
|  | Urban residence | - | - | - | - | - | 1 | - | 1 | 1 |
|  | Employment/  occupation type | 1 | - | - | 1 | - | 2 | - | 4 | 3 |
|  | Low income/on financial assistance | - | - | - | - | 1 | 2 | - | 3 | 2 |
|  | At least 1 factor within category | ✓ | - | - | ✓ | ✓ | ✓ | - | - | 4 |
| **Social support**  **(n = 15)** | Lack of husband support | - | 1 | 1 | 3 | - | 1 | - | 6 | 4 |
|  | Lack of support (family/friend) | - | - | - | 4 | 1 | - | - | 5 | 2 |
|  | Lack of companion to go | - | - | - | - | 3 | - | - | 3 | 1 |
|  | Living arrangement | - | - | - | 1 | - | - | - | 1 | 1 |
|  | At least 1 factor within category | - | ✓ | ✓ | ✓ | ✓ | ✓ | - | - | 5 |
| **Psychological, emotional**  **(n = 43)** | Fear of pain | - | 1 | 3* | 8 | 3 | 8 | - | 23 | 5 |
|  | Fear of result | - | 2* | 2 | 5 | 4 | 6 | - | 19 | 5 |
|  | Fear (general) | - | 1 | - | 6 | 1 | 4 | - | 12 | 4 |
|  | Fear of side effects | - | - | - | - | 1 | 1 | - | 2 | 3 |
|  | Worry | - | - | - | 3 | - | - | - | 3 | 1 |
|  | Embarrassed | - | 2* | 3* | 12* | 4 | 12* | - | 33 | 5 |
|  | Concern about equipment | - | - | - | 1 | - | 1 | - | 2 | 2 |
|  | Concern about improper sampling | - | - | - | 1 | - | - | - | 1 | 1 |
|  | Not comfortable with procedure | - | - | - | 2 | - | - | - | 2 | 1 |
|  | Not ready/hesitancy | - | - | - | 1 | - | 1 | - | 2 | 2 |
|  | At least 1 factor within category | - | ✓ | ✓ | ✓ | ✓ | ✓ | - | - | 5 |
| **Knowledge**  **(n = 38)** | Poor knowledge (disease) | - | 2* | - | - | - | - | - | 2 | 1 |
|  | Poor knowledge of screening | - | 2* | 1 | 9* | 4 | 11* | - | 27 | 5 |
|  | Poor HPV knowledge | - | - | - | - | - | 1 | - | 1 | ✓ |
|  | Poor awareness | - | 1 | 2 | 5 | 3 | 1 | 2 | 14 | 6 |
|  | At least 1 factor within category | - | ✓ | ✓ | ✓ | ✓ | ✓ | ✓ | - | 6 |
| **Risk perception**  **(n = 35)** | Perceived susceptibility | - | 1 | 2 | 6 | 7* | 8 | - | 24 | 5 |
|  | Not at risk | - | 1 | 2 | - | 3 | 4 | - | 10 | 4 |
|  | No symptoms | - | 2* | 2 | 4 | - | 11* | 1 | 20 | 5 |
|  | At least 1 factor within category | - | ✓ | ✓ | ✓ | ✓ | ✓ | ✓ | - | 6 |
| **Perception, attitude, belief**  **(n = 20)** | Belief towards screening/disease | - | - | 1 | 4 | 3 | 1 | - | 9 | 4 |
|  | Poor attitude (test/general) | - | 1 | - | 1 | - | 2 | - | 4 | 3 |
|  | Fatalistic attitude | - | 1 | - | - | 4 | - | - | 5 | 2 |
|  | High perceived barriers | - | 1 | - | - | - | 1 | - | 2 | 2 |
|  | Poor self-perception (test/disease) | - | 1 | - | - | - | - | - | 1 | 1 |
|  | High perceived behavior control | - | - | - | - | 1 | - | - | 1 | 1 |
|  | Cultural | - | - | - | 1 | - | 1 | - | 2 | 2 |
|  | At least 1 factor within category | - | ✓ | ✓ | ✓ | ✓ | ✓ | - | - | 5 |
| **Motivation, preference**  **(n = 29)** | Low motivation | - | 1 | - | - | - | - | - | 1 | 1 |
|  | Dislike test that are painful/invasive | - | - | - | 1 | 1 | - | - | 2 | 2 |
|  | Inconvenience | - | - | - | - | 3 | 1 | - | 4 | 2 |
|  | Busy/no time | - | - | 1 | 11* | 6* | 9 | - | 27 | 4 |
|  | At least 1 factor within category | - | ✓ | ✓ | ✓ | ✓ | ✓ | - | - | 5 |
| **Financial access**  **(n = 23)** | Cost (screening, transport, treatment) | - | 1 | 3* | 6 | 6* | 6 | - | 22 | 5 |
|  | Lack of insurance | - | - | - | - | - | 1 | - | 1 | 2 |
|  | Financial problem | - | - | - | - | - | 1 | - | 1 | 1 |
|  | At least 1 factor within category | - | ✓ | ✓ | ✓ | ✓ | ✓ | - | - | 5 |
| **Health system**  **(n = 31)** | Health center characteristics (manpower, location, operations) | - | 1 | 2 | 8 | 3 | 4 | - | 18 | 5 |
|  | Limited promotion/community mobilization | - | 1 | - | - | - | 1 | - | 2 | 2 |
|  | Delay in test result/procedure | - | - | - | 1 | 1 | 1 | - | 3 | 4 |
|  | Not prescribed/no healthcare worker advice | - | - | 2 | 2 | 1 | - | 1 | 6 | 4 |
|  | Poor healthcare worker/system impression | - | - | - | 2 | 1 | 5 | 1 | 9 | 4 |
|  | Low disease priority (nation) | - | - | - | 1 | - | - | - | 1 | 1 |
|  | Inadequate government resources | - | - | - | 1 | - | - | - | 1 | 1 |
|  | Passive follow up system | - | - | - | 1 | - | - | - | 1 | 1 |
|  | At least 1 factor within category | - | ✓ | ✓ | ✓ | ✓ | ✓ | ✓ | - | 6 |
| **Others**  **(n = 15)** | Do not attend breast examination | 1 | - | - | - | - | - | - | 1 | 1 |
|  | Just married | - | - | - | 1 | - | - | - | 1 | 1 |
|  | Post menopause | - | - | - | - | 1 | 1 | - | 2 | 2 |
|  | Post hysterectomy | - | - | - | - | - | 1 | - | 1 | 1 |
|  | Have not had sex | - | - | 2 | 2 | 2 | 2 | - | 8 | 4 |
|  | Using contraceptives | - | - | - | 2 | - | - | - | 2 | 1 |
|  | Type of contraceptive | - | - | - | 1 | - | - | - | 1 | 1 |
|  | At least 1 factor within category | ✓ | - | ✓ | ✓ | ✓ | ✓ | - | - | 5 |

*top three barriers by publication number for Malaysia, Indonesia, Laos, Singapore, and Thailand
