# Supplementary material for: Barriers to and Facilitators of Cervical Cancer Screening among Women in Southeast Asia: A Systematic Review
Source: Int J Environ Res Public Health. 2021 Apr 26;18(9):4586. doi: 10.3390/ijerph18094586 (PMC8123618; doi:10.3390/ijerph18094586)
Supplement: Supplementary file 1 [file ijerph-18-04586-s001.zip › Table S4 Facilitator to screening.docx]

**Table S4.** Facilitators of cervical cancer screening in Southeast Asia

| **Categories** | **Facilitators** | **Brunei  (n = 1)** | **Cambodia  (n = 1)** | **Indonesia  (n = 13)** | **Laos  (n = 1)** | **Malaysia  (n = 26)** | **Myanmar (n = 1)** | **Singapore  (n = 12)** | **Thailand  (n = 17)** | **Vietnam  (n = 1)** | **Total**  **number of studies** | **Total number of countries** |
| --- | --- | --- | --- | --- | --- | --- | --- | --- | --- | --- | --- | --- |
| **Demographics**  **(n = 33)** | Age | - | 1 | 1 | - | 10* | 1 | 3* | 5* | - | 21 | 6 |
|  | Age of first intercourse | - | - | - | - | - | - | - | 1 | - | 1 | 1 |
|  | Married | 1 | - | - | - | 6* | - | - | 2 | - | 9 | 3 |
|  | Ethnicity | - | - | - | - | 2 | - | 1 | - | - | 3 | 2 |
|  | Have comorbidities | 1 | - | - | - | 1 | - | 1 | 1 | - | 4 | 4 |
|  | Higher Charlson Comorbidity Index | - | - | 1 | - | - | - | - | - | - | 1 | 1 |
|  | History of sexually transmitted disease | - | - | - | - | - | - | - | 1 | - | 1 | 1 |
|  | Presence of family comorbidity | 1 | - | - | - | - | - | - | - | - | 1 | 1 |
|  | Family history of cancer | - | - | - | - | 1 | 1 | - | 1 | - | 3 | 3 |
|  | Religion | - | - | - | - | 1 | - | - | - | - | 1 | 1 |
|  | Parity (higher) | - | - | 1 | - | 5 | - | - | 3 | - | 9 | 3 |
|  | Have young children | - | - | - | - | 1 | - | - | - | - | 1 | 1 |
|  | At least 1 factor within category | ✓ | ✓ | ✓ | - | ✓ | ✓ | ✓ | ✓ | - | - | 7 |
| **Socio-economic**  **(n = 19)** | Education (higher) | - | - | 2* | - | 4 | - | 1 | 4 | - | 11 | 4 |
|  | Region of residence | - | - | - | - | - | - | - | 1 | - | 1 | 1 |
|  | Rural house | - | - | - | - | 1 | - | - | - | - | 1 | 1 |
|  | Occupation type | - | - | - | - | - | - | - | 2 | - | 2 | 1 |
|  | Employed | - | - | - | - | - | - | 1 | - | - | 1 | 1 |
|  | Longer duration of work (years) | - | - | - | - | 1 | - | - | - | - | 1 | 1 |
|  | Income (high) | - | - | 2* | - | 3 | - | 1 | 4 | - | 10 | 4 |
|  | On subsidized care | - | - | - | - | - | - | 1 | - | - | 1 | 1 |
|  | At least 1 factor within category | - | - | ✓ | - | ✓ | - | ✓ | ✓ | - | - | 4 |
| **Healthcare utilization**  **(n = 14)** | Have annual checkup/regular physician | - | - | - | - | 2 | - | 1 | 1 | - | 4 | 3 |
|  | Part of routine examination | - | - | - | - | 1 | - | 1 | 4 | - | 6 | 3 |
|  | Health check done in 3-5 years | - | - | - | - | 1 | - | 1 | - | - | 2 | 2 |
|  | Breast cancer screening | - | - | - | - | 2 | - | - | - | - | 2 | 1 |
|  | Characteristics of last smear | - | - | - | - | 1 | - | 1 | - | - | 2 | 2 |
|  | At least 1 factor within category | - | - | - | - | ✓ | - | ✓ | ✓ | - | - | 3 |
| **Social support**  **(n = 14)** | Support from husband | - | - | - | - | 3 | - | 1 | 1 | 1 | 6 | 4 |
|  | Support from friends/family | - | - | 3* | - | 2 | - | 3* | 1 | - | 9 | 4 |
|  | At least 1 factor within category | - | - | ✓ | - | ✓ | - | ✓ | ✓ | ✓ | - | 5 |
| **Psychological, emotional**  **(n = 5)** | Relieved after test | - | - | - | - | 1 | - | - | - | - | 1 | 1 |
|  | Suspicion of cancer | - | - | - | - | 1 | - | - | 2 | - | 3 | 2 |
|  | Fear (of cancer) | - | - | - | - | - | - | - | 2 | - | 2 | 1 |
|  | At least 1 factor within category | - | - | - | - | ✓ | - | - | ✓ | - | - | 2 |
| **Knowledge**  **(n = 23)** | Good knowledge (unspecific) | - | - | 2* | - | - | - | - | 1 | - | 3 | 2 |
|  | Good knowledge (screening) | - | - | - | - | 5 | - | - | - | - | 5 | 1 |
|  | Good knowledge (disease) | - | - | - | - | 2 | - | - | - | - | 2 | 1 |
|  | Good knowledge (disease and test) | - | - | - | - | 2 | 1 | 2 | 1 | - | 6 | 4 |
|  | Good knowledge (reproductive health) | - | - | - | - | 1 | - | - | - | - | 1 | 1 |
|  | Good awareness of HPV risk | - | - | - | - | 1 | - | - | - | - | 1 | 1 |
|  | Good awareness | - | 1 | 1 | - | 1 | - | 3* | 1 | - | 7 | 5 |
|  | At least 1 factor within category | - | ✓ | ✓ | - | ✓ | ✓ | ✓ | ✓ | - | - | 6 |
| **Risk perception**  **(n = 11)** | Perceived susceptibility | - | - | - | - | 1 | - | 1 | 1 | - | 3 | 3 |
|  | Have symptoms | - | - | - | - | 1 | - | 1 | 5* | - | 7 | 3 |
|  | High perceived threat | - | - | 1 | - | - | - | - | - | - | 1 | 1 |
|  | At least 1 factor within category | - | - | ✓ | - | ✓ | - | ✓ | ✓ | - | - | 4 |
| **Perception, attitude, belief**  **(n = 29)** | Belief in screening/disease | - | - | 1 | - | 1 | - | 1 | 1 | - | 4 | 4 |
|  | Good attitude | - | - | 2* | - | 5 | - | - | 3 | - | 10 | 3 |
|  | Low barrier perception | - | - | - | - | 1 | 1 | 1 | 2 | - | 5 | 4 |
|  | Perception of behavior control | - | - | 1 | - | - | - | - | 1 | - | 2 | 2 |
|  | Perceived benefit | - | - | 1 | - | 2 | - | 3* | - | - | 6 | 3 |
|  | Perception (unspecified) | - | - | 1 | - | - | - | - | - | - | 1 | 1 |
|  | Perception of control over life and environment | - | - | 1 | - | - | - | - | - | - | 1 | 1 |
|  | Perception social pressure to obtain test | - | - | - | - | - | - | 1 | 1 | - | 2 | 2 |
|  | Behavior | - | - | 1 | - | - | - | - | - | - | 1 | 1 |
|  | At least 1 factor within category | - | - | ✓ | - | ✓ | ✓ | ✓ | ✓ | - | - | 5 |
| **Motivation, preference**  **(n = 8)** | Motivation | - | - | 1 | - | - | - | - | - | - | 1 | 1 |
|  | Desire to know more/curiosity | - | - | 1 | - | - | - | - | 1 | - | 2 | 2 |
|  | Intention | - | - | 3* | 1 | - | - | - | - | - | 4 | 2 |
|  | Positive past experience | - | - | - | - | - | - | 1 | - | - | 1 | 1 |
|  | At least 1 factor within category | - | - | ✓ | ✓ | - | - | ✓ | ✓ | - | - | 4 |
| **Financial access**  **(n = 11)** | Low cost/free test | - | - | 1 | - | - | - | 2 | 1 | 1 | 5 | 4 |
|  | Test expensive | - | - | - | - | - | - | - | 1 | - | 1 | 1 |
|  | Have insurance | - | - | 1 | - | 1 | - | - | 1 | - | 3 | 3 |
|  | Insurance type (civil servant vs basic) | - | - | - | - | - | - | - | 1 | - | 1 | 1 |
|  | Willingness to pay for screening | - | - | - | - | - | 1 | - | - | - | 1 | 1 |
|  | At least 1 factor within category | - | - | ✓ | - | ✓ | ✓ | ✓ | ✓ | ✓ | - | 6 |
| **Health system**  **(n = 27)** | Health center characteristics (manpower, location, operation) | - | - | 2* | - | - | - | 3* | 1 | - | 6 | 3 |
|  | Healthcare worker advice | - | - | - | - | 6* | 1 | 3* | 6* | 1 | 17 | 5 |
|  | Government/employers advice | - | - | - | - | 1 | - | - | 3 | - | 4 | 2 |
|  | Reminder letter for patient | - | - | - | - | - | - | 1 | - | - | 1 | 1 |
|  | Reminder for healthcare provider | - | - | - | - | - | - | 1 | 1 | - | 2 | 2 |
|  | Insufficient disease information from healthcare worker | - | - | - | - | 1 | - | - | - | - | 1 | 1 |
|  | Organized screening/community outreach | - | - | 2* | - | 1 | - | 1 | 1 | - | 5 | 4 |
|  | At least 1 factor within category | - | - | ✓ | - | ✓ | ✓ | ✓ | ✓ | ✓ | - | 6 |
| **Others**  **(n = 10)** | Diet | - | - | - | - | - | - | - | 1 | - | 1 | 1 |
|  | No alcohol | 1 | - | - | - | - | - | - | - | - | 1 | 1 |
|  | Higher CD4 count | - | - | - | - | - | - | - | 1 | - | 1 | 1 |
|  | On hormonal therapy | - | - | - | - | - | - | - | 1 | - | 1 | 1 |
|  | On contraceptive | - | - | - | - | 5 | - | - | - | - | 5 | 1 |
|  | Practice of screening | - | - | - | - | 2 | - | - | - | - | 2 | 1 |
|  | At least 1 factor within category | ✓ | - | - | - | ✓ | - | - | ✓ | - | - | 3 |

*top three facilitators by publication number for Malaysia, Indonesia, Singapore, and Thailand
