# Supplementary figures and images for: Barriers to and Facilitators of Cervical Cancer Screening among Women in Southeast Asia: A Systematic Review
Source: Int J Environ Res Public Health. 2021 Apr 26;18(9):4586. doi: 10.3390/ijerph18094586 (PMC8123618; doi:10.3390/ijerph18094586)

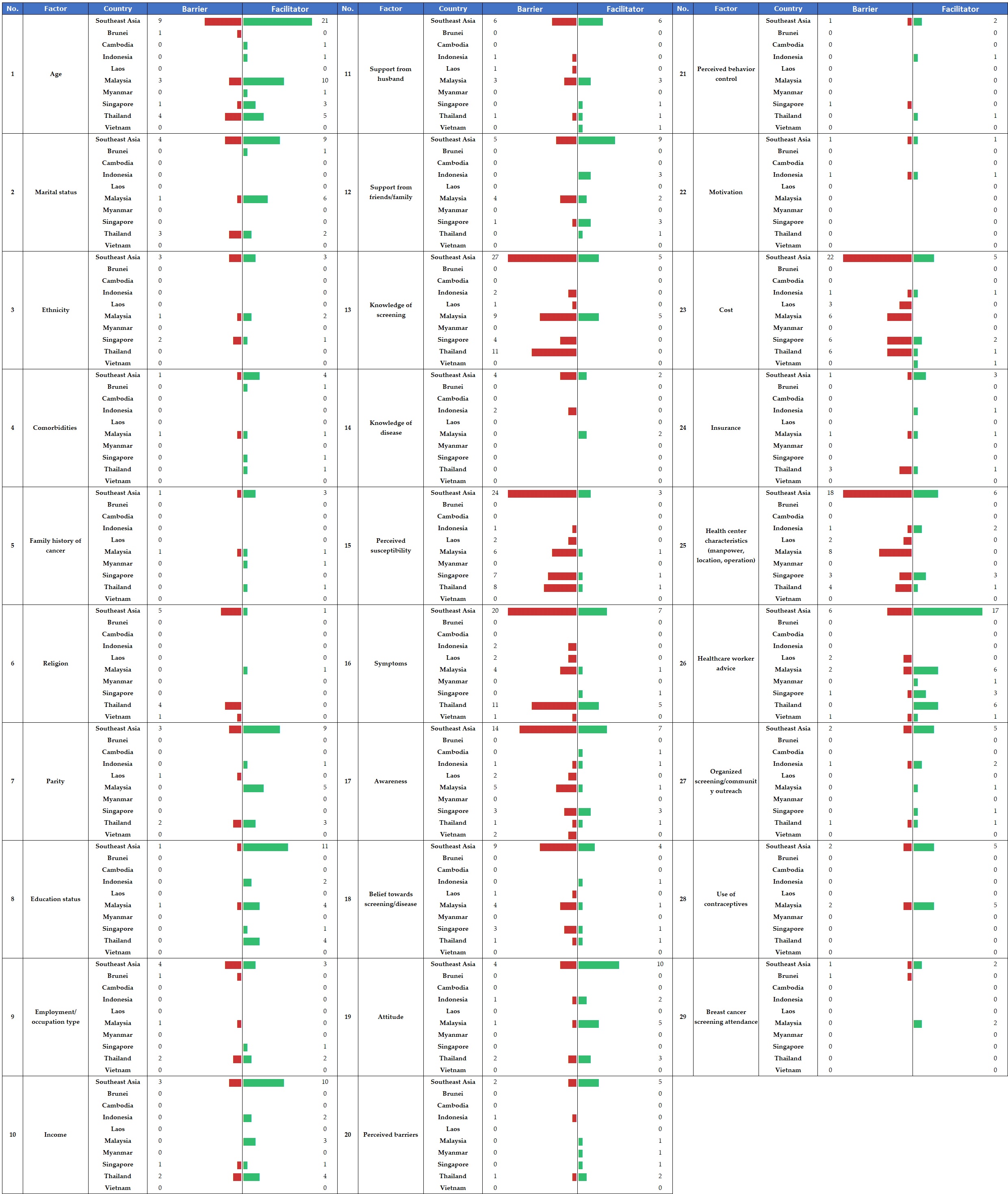

Supplement: Supplementary file 1 [file ijerph-18-04586-s001.zip › Figure S1 Factors described as barriers to and facilitators of cervical cancer screening.jpg]
